# Preparation of Crosslinked Chitosan/TiO_2_ Composite Beads for Photocatalytic Removal of Reactive Black 5: Effect of Material Composition and Process Parameters on Decolorization and Mineralization

**DOI:** 10.3390/polym18161975

**Published:** 2026-08-13

**Authors:** Nuri Bozkurt, Şeyda Taşar, Gamze Sak, Gülbeyi Dursun

**Affiliations:** Department of Chemical Engineering, Faculty of Engineering, Fırat University, 23119 Elazığ, Türkiye

**Keywords:** chitosan, titanium dioxide, composite beads, photocatalysis, reactive black 5, wastewater treatment

## Abstract

The immobilization of photocatalysts onto biodegradable polymeric supports has emerged as an effective strategy to overcome catalyst recovery limitations associated with conventional slurry photocatalytic systems. In this study, chitosan/TiO_2_ composite beads with different chitosan properties and TiO_2_ loadings were synthesized and evaluated for the photocatalytic removal of Reactive Black 5 (RB5), a recalcitrant azo dye commonly encountered in textile wastewater. The effects of chitosan molecular weight, degree of deacetylation, and crosslinking treatment on the structural characteristics and photocatalytic performance of the composite beads were systematically investigated. The synthesized composites were characterized through physical property measurements, point of zero charge (pH_pzc_) determination, and FTIR analyses. Photocatalytic performance was evaluated under various operational conditions, including pH, catalyst dosage, initial dye concentration, and temperature. Among the prepared materials, the crosslinked chitosan/TiO_2_ composite bead produced from chitosan with an 85% degree of deacetylation exhibited the highest mineralization efficiency, achieving 76.23% total organic carbon (TOC) removal. FTIR analyses performed before and after treatment indicated that RB5 removal occurred through the combined effects of adsorption and photocatalytic oxidation. The effects of operational parameters revealed that acidic conditions significantly enhanced RB5 removal, while increasing temperature improved reaction kinetics and overall degradation efficiency. Kinetic studies indicated that the photocatalytic degradation process was best described by the pseudo-first-order kinetic model, with correlation coefficients ranging from 0.9841 to 0.9976. Arrhenius analysis yielded an apparent activation energy of 11.76 kJ mol^−1^, indicating a low energy barrier for the degradation process. The results demonstrate that crosslinked chitosan/TiO_2_ composite beads are promising, environmentally friendly, and sustainable photocatalytic materials for the treatment of dye-containing wastewater and advanced water purification applications.

## 1. Introduction

The rapid expansion of industrial activities has led to the generation of large volumes of wastewater containing complex organic pollutants, among which synthetic dyes represent one of the most significant environmental concerns. Textile, paper, leather, printing, and dye-manufacturing industries release considerable quantities of colored effluents into aquatic environments, adversely affecting water quality and ecosystem health. The presence of dyes in receiving waters reduces light penetration, inhibits photosynthetic activity, decreases dissolved oxygen levels, and may induce toxic, mutagenic, and carcinogenic effects in aquatic organisms and humans [1,2,3]. Consequently, the development of efficient, sustainable, and environmentally friendly technologies for the treatment of dye-containing wastewater has become an important research priority in accordance with global sustainability goals and water protection strategies [1].

Among the numerous classes of synthetic dyes, azo dyes constitute the largest and most widely used group in industrial applications. These compounds are characterized by the presence of one or more azo (–N=N–) bonds linking aromatic structures that provide excellent color stability and resistance to chemical and biological degradation. Reactive Black 5 (RB5), an anionic diazo dye containing azo and sulfonate groups, is extensively used in textile dyeing processes because of its high water solubility, color fastness, and low manufacturing cost. In addition, RB5 is frequently employed as a model pollutant in adsorption and photocatalytic studies due to its complex molecular structure, high chemical stability, low biodegradability, and resistance to conventional treatment methods. The presence of multiple aromatic rings and sulfonate groups makes RB5 particularly difficult to remove from aqueous environments and represents a challenging target pollutant for evaluating the effectiveness of newly developed treatment materials. However, the complex aromatic structure and high stability of RB5 make it resistant to conventional treatment processes, leading to its persistence in aquatic environments [4,5,6]. The incomplete degradation of azo dyes may also generate toxic aromatic amines that pose additional environmental and health risks. Therefore, effective removal and mineralization of RB5 remain critical challenges in wastewater treatment technologies [2,7].

Conventional treatment methods such as coagulation–flocculation, adsorption, membrane filtration, ion exchange, and biological treatment have been widely applied for dye removal. Although these methods can reduce dye concentrations to some extent, they frequently suffer from limitations including incomplete mineralization, secondary sludge generation, membrane fouling, high chemical consumption, and elevated operational costs [3,7]. Adsorption processes, for example, transfer pollutants from one phase to another without destroying their chemical structure, whereas biological treatment systems often exhibit limited efficiency toward recalcitrant azo compounds [8,9]. These limitations have stimulated growing interest in advanced oxidation processes (AOPs), which are capable of generating highly reactive oxidizing species that can degrade complex organic pollutants into less harmful or completely mineralized products [2,3,7].

Among the various AOPs, heterogeneous photocatalysis has emerged as one of the most promising approaches for the treatment of dye-containing wastewater. Photocatalytic processes involve the activation of semiconductor materials by light irradiation, resulting in the generation of electron–hole pairs capable of producing reactive oxygen species (ROS), such as hydroxyl radicals (•OH) and superoxide radicals (O_2_•^−^). These highly reactive species attack organic pollutants and progressively oxidize them into carbon dioxide, water, and inorganic ions [2,7,10,11]. Numerous studies have indicated the effectiveness of photocatalytic systems for the degradation of textile dyes, phenolic compounds, pharmaceuticals, and various emerging contaminants [4,12].

Titanium dioxide (TiO_2_) is the most extensively studied photocatalyst because of its high photocatalytic activity, chemical stability, low toxicity, affordability, and resistance to photocorrosion [2,11,13]. Upon UV irradiation, electrons are excited from the valence band to the conduction band, leaving positively charged holes that participate in oxidation reactions. The generated electron–hole pairs react with water and dissolved oxygen molecules to produce ROS responsible for contaminant degradation [10,11]. TiO_2_ exists mainly in anatase, rutile, and brookite crystalline forms, with anatase generally exhibiting superior photocatalytic activity due to its favorable charge carrier dynamics and lower recombination rate [11]. Various modification strategies, including metal doping, non-metal doping, and composite formation, have been proposed to further enhance the photocatalytic efficiency of TiO_2_ [13,14,15].

Despite its outstanding photocatalytic properties, the practical application of TiO_2_ nanoparticles remains restricted by several drawbacks. The nanoscale dimensions of TiO_2_ particles complicate catalyst recovery after treatment, increase operational costs, and limit large-scale implementation. Furthermore, TiO_2_ nanoparticles tend to aggregate in aqueous media, reducing active surface area and photocatalytic efficiency [15,16,17]. The immobilization of TiO_2_ onto suitable support materials has therefore attracted considerable attention as an effective strategy to overcome these limitations while improving catalyst stability and reusability [18,19,20]. In particular, immobilization of photocatalysts in bead form provides several practical advantages, including easier catalyst recovery, reduced nanoparticle release into treated water, lower pressure drops in packed-bed systems, and improved suitability for continuous-flow reactor applications. These advantages significantly enhance the feasibility of large-scale wastewater treatment processes and facilitate photocatalyst reuse without requiring complex separation operations.

Natural biopolymers have emerged as attractive support materials for photocatalyst immobilization because of their biodegradability, environmental compatibility, and abundance. Among these materials, chitosan has received particular attention owing to its unique physicochemical and biological properties [21,22]. Chitosan is a naturally occurring polysaccharide obtained through the deacetylation of chitin, the second most abundant biopolymer on Earth after cellulose [21,22,23]. It consists primarily of β-(1-4)-D-glucosamine and N-acetyl-D-glucosamine units and contains abundant amino and hydroxyl functional groups that provide high chemical reactivity and adsorption capacity [22,23,24]. Chitosan is biodegradable, biocompatible, non-toxic, antimicrobial, and capable of forming films, hydrogels, membranes, nanoparticles, and bead structures [25,26,27,28,29]. Furthermore, chitosan has been successfully utilized for the synthesis of nano- and microstructured materials due to its excellent film-forming ability, functional amino groups, and inherent antimicrobial properties, making it an attractive material for environmental and biomedical applications [30]. Similarly, surface-engineered nanoparticles have attracted considerable attention because of their tunable physicochemical properties, enhanced functionality, and broad applicability in environmental and biomedical fields [31].

The physicochemical properties of chitosan are strongly influenced by its molecular weight and degree of deacetylation (DD) [32]. These parameters affect solubility, charge density, swelling behavior, adsorption capacity, mechanical strength, and biological activity [24,26,33,34]. Under acidic conditions, the amino groups of chitosan become protonated, generating positively charged sites capable of attracting negatively charged pollutants through electrostatic interactions [8,9,35]. This characteristic makes chitosan particularly attractive for the removal of anionic dyes from aqueous solutions. Numerous studies have indicated the excellent adsorption performance of chitosan-based materials toward various dye molecules and organic contaminants [8,9,21,36,37]. Previous studies have also indicated the potential of chitosan-based materials for environmental remediation. Demirtaş et al. [36,38] reported efficient removal of Basic Blue 41 dye using crosslinked chitosan microspheres and chitosan-based particles, highlighting the significant influence of particle structure on sorption performance. Furthermore, İpek et al. [37]. developed magnetic field-sensitive chitosan particles for dye removal from aqueous solutions and indicated enhanced adsorption efficiency and operational flexibility. In addition to adsorption-based applications, recent photocatalytic studies conducted by Dursun and co-workers revealed the successful degradation of azo dyes and phenolic pollutants using TiO_2_ and ZnO-based photocatalytic systems under UV irradiation [39,40]. These findings indicate that combining the adsorption capability of chitosan with the oxidative potential of semiconductor photocatalysts represents a promising strategy for the efficient treatment of dye-containing wastewaters.

The incorporation of TiO_2_ into chitosan matrices enables the development of multifunctional composite materials that combine adsorption and photocatalytic degradation mechanisms [41]. In these systems, chitosan serves as both a support matrix and an adsorbent, concentrating pollutant molecules near photocatalytically active TiO_2_ sites. Simultaneously, the polymer matrix helps prevent TiO_2_ aggregation, improves nanoparticle dispersion, and facilitates catalyst recovery after treatment [16,42]. FTIR studies have revealed strong interactions between chitosan and TiO_2_ through hydrogen bonding and other intermolecular forces, contributing to improved structural stability and enhanced photocatalytic performance [10,42,43].

Recent studies have reported promising applications of chitosan/TiO_2_ composites in environmental remediation. Habiba et al. [44] indicated efficient Congo Red adsorption using chitosan–TiO_2_ nanocomposites, while Zioui et al. [10] reported successful tartrazine degradation using TiO_2_–chitosan composite films. Indira et al. [14] showed that Ce-doped TiO_2_/chitosan nanocomposites achieved approximately 95% degradation of Rhodamine B, significantly outperforming TiO_2_. Similarly, Afzal et al. [16] reported complete methyl orange removal under optimized conditions using chitosan-modified TiO_2_ composites. Hoang and Nguyen [42] observed enhanced degradation kinetics for industrial dyes using chitosan/TiO_2_/glycerol films, whereas Saefumillah et al. [18] achieved dye removal efficiencies exceeding 90% using TiO_2_–chitosan immobilized on glass beads in both batch and flow systems. More recently, Aoudjit et al. [20] indicated effective methyl orange degradation using TiO_2_ immobilized within chitosan matrices under solar irradiation. These findings confirm the significant potential of chitosan/TiO_2_ composites as sustainable photocatalytic materials.

Although considerable progress has been achieved in the development of chitosan/TiO_2_ composites, several important knowledge gaps remain. Most previous studies have focused on films, membranes, coatings, or nanoparticle systems, while comparatively fewer investigations have examined bead-type photocatalysts that offer easier separation and reuse. Furthermore, systematic evaluations of the effects of chitosan molecular weight, degree of deacetylation, TiO_2_ loading ratio, and crosslinking treatment on photocatalytic degradation and mineralization performance remain limited. The influence of these material parameters on adsorption–photocatalysis synergy, surface charge characteristics, and degradation kinetics has not yet been comprehensively established for Reactive Black 5 removal [16,18,20,42].

Therefore, the objective of the present study was to synthesize crosslinked chitosan/TiO_2_ composite beads with different chitosan characteristics and TiO_2_ contents and to evaluate their applicability for the photocatalytic removal of Reactive Black 5 from aqueous solutions. The prepared composite beads were characterized through physicochemical analyses, FTIR spectroscopy, and point of zero charge (pH_pzc_) determination. The effects of operational parameters, including solution pH, catalyst dosage, initial dye concentration, and temperature, were systematically investigated. In addition, total organic carbon (TOC) analyses were performed to assess mineralization efficiency, while kinetic and Arrhenius models were employed to evaluate reaction mechanisms and temperature dependence. The findings provide valuable insights into the design of sustainable, biodegradable, and highly efficient chitosan/TiO_2_ photocatalytic materials for advanced wastewater treatment applications.

## 2. Materials and Methods

### 2.1. Materials

Chitosan samples with different molecular weights and degrees of deacetylation were used as the polymer matrix for the preparation of composite beads. Low molecular weight (LMW), medium molecular weight (MMW), and high molecular weight (HMW) chitosan samples (degree of deacetylation ≥ 85%) were supplied by Sigma-Aldrich (Burlington, MA, USA). Commercial chitosan with a degree of deacetylation of approximately 75% was obtained from Adaga Kimya (Antalya, Türkiye). Titanium dioxide (TiO_2_, anatase phase, purity ≥ 80%) was used as the photocatalytic component (Evonik Industries, Essen, Germany). Acetic acid, sodium hydroxide (NaOH), and all other analytical-grade chemicals were purchased from Sigma-Aldrich (St. Louis, MO, USA) and used without further purification. Reactive Black 5 (RB5) was selected as the model pollutant and all solutions were prepared using deionized water.

### 2.2. Preparation of Chitosan/TiO_2_ Composite Beads

Chitosan solutions were prepared by dissolving 1.0 g of chitosan in 100 mL of 1% (*v*/*v*) acetic acid solution under magnetic stirring at 900 rpm for 6 h at room temperature. TiO_2_ nanoparticles were subsequently incorporated into the chitosan solution at different chitosan/TiO_2_ mass ratios (1/1, 1/2, 2/1, 1/3, and 3/1). The resulting suspensions were stirred at 900 rpm for 60 min and then subjected to ultrasonic treatment (20 kHz; BANDELIN electronic GmbH & Co. KG, Berlin, Germany) for 10 min to ensure homogeneous dispersion of TiO_2_ particles.

**Figure 1:**
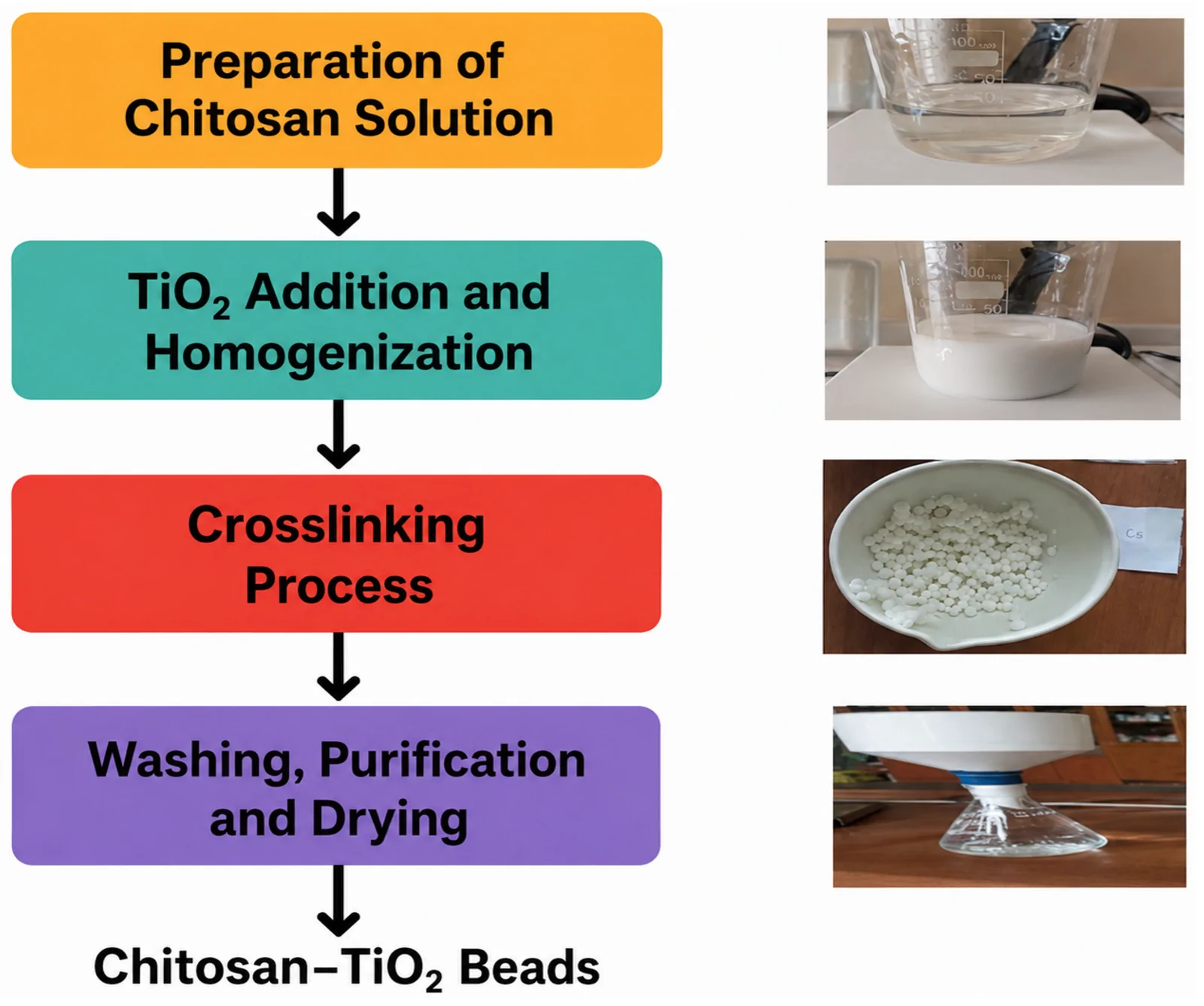
Schematic illustration of the preparation procedure of chitosan/TiO_2_ composite beads. (The layout of this figure was prepared with the assistance of ChatGPT 5.6 (OpenAI) solely for graphical presentation purposes).

Composite beads were produced by dropping the chitosan/TiO_2_ suspension into a NaOH coagulation bath using a peristaltic dosing system at a flow rate corresponding to approximately three drops per minute. Two NaOH concentrations (20 and 40 g L^−1^) were evaluated during preliminary studies. The formed beads were kept in the coagulation bath until complete gelation was achieved, washed repeatedly with deionized water until neutral pH was reached, and subsequently stored in deionized water prior to use. For selected formulations, a crosslinking treatment was applied to improve structural stability. The obtained composite beads were coded according to chitosan characteristics, TiO_2_ content, and crosslinking condition (Figure 1). The particle production method employed in this study has been successfully utilized in previous studies conducted by our research group for the preparation of chitosan-based sorbent particles possessing different molecular weights and degrees of deacetylation [36,38,45].

### 2.3. Characterization of Composite Beads

The physical properties of the prepared beads were evaluated by determining particle diameter, volume, density, and average particle weight. The point of zero charge (pH_pzc_) of the selected composite beads was determined using the pH drift method [46].

Fourier Transform Infrared Spectroscopy (FTIR) analyses were performed using a Shimadzu IRSpirit spectrometer (Kyoto, Japan) within the range of 4000–400 cm^−1^ with 45 scans per sample. FTIR spectra were recorded before and after photocatalytic experiments to evaluate structural changes and identify possible interactions between RB5 molecules and the composite surface.

### 2.4. Photocatalytic Experiments

**Figure 2:**
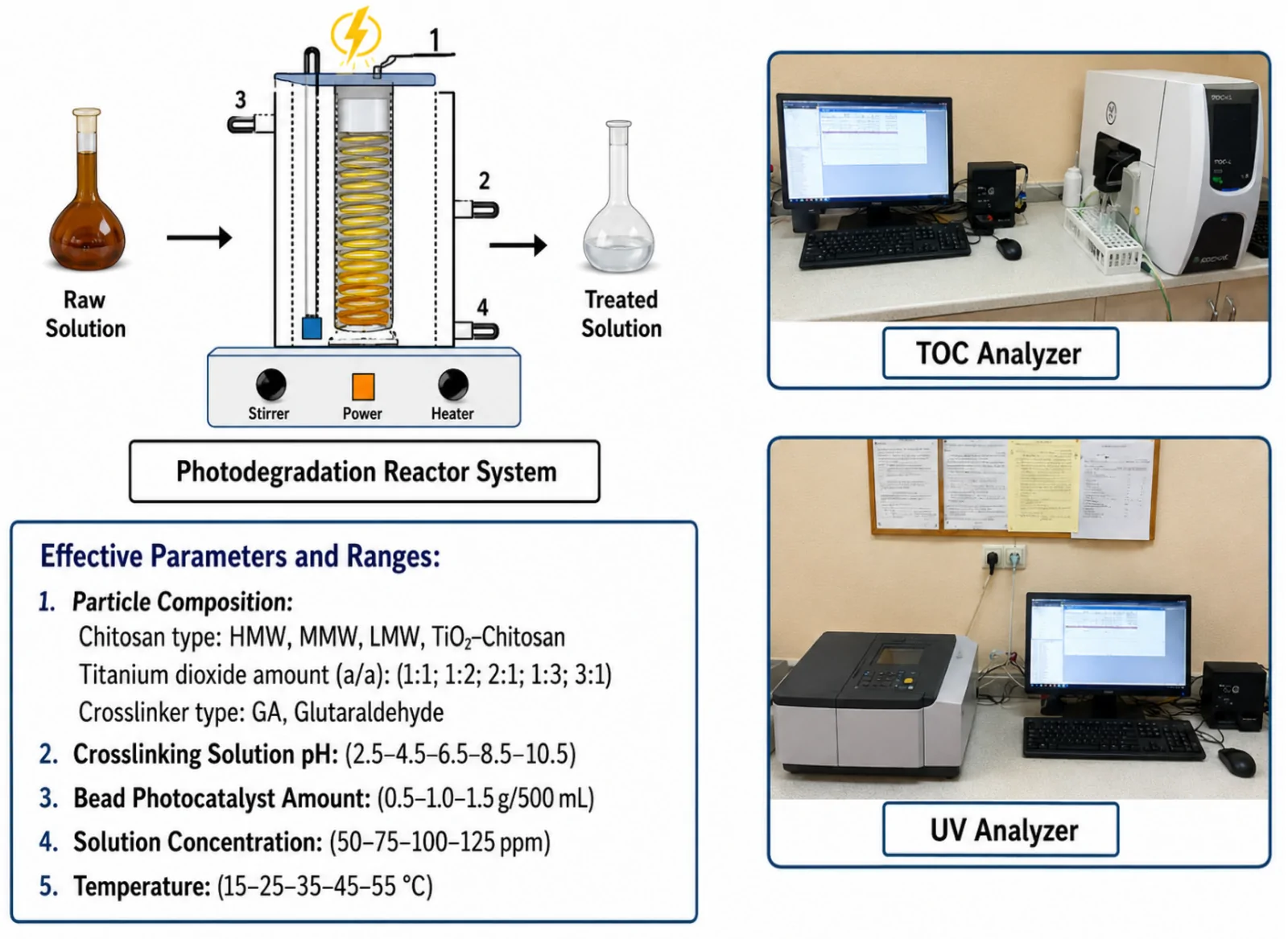
Schematic representation of photocatalytic degradation and analytical procedure for organic pollutant removal from aqueous solution (The layout of this figure was prepared with the assistance of ChatGPT 5.6 (OpenAI) solely for graphical presentation purposes).

Photocatalytic degradation experiments were carried out in a cylindrical jacketed glass reactor with a total volume of 500 mL. UV irradiation was provided by a 21 W UV-C lamp (280 nm) positioned centrally within the reactor (Figure 2). Continuous mixing was maintained at 300 rpm using a mechanical stirrer, while compressed air was supplied through a diffuser at a flow rate of 0.02 L min^−1^. The reactor temperature was controlled by circulating water through the external jacket. RB5 solutions of different concentrations were prepared and treated using the synthesized composite beads. Samples were collected at predetermined time intervals and filtered prior to analysis. The residual RB5 concentration was determined using a UV–Vis spectrophotometer (Shimadzu Corporation, Kyoto, Japan) at the maximum absorption wavelength of 597 nm. The photocatalytic reactor system used in this study has been successfully applied in previous studies conducted by our research group for the degradation of various organic pollutants in aqueous solutions using semiconductor photocatalysts, including TiO_2_ and ZnO [39,40,47].

The effects of operational parameters including initial pH (2.5–10.5), composite bead dosage (0.5–1.5 g), initial RB5 concentration (50–125 mg L^−1^), and temperature (15–55 °C) were systematically investigated. The dye removal efficiency was calculated according to Equation (1):

(1) $$Yield\;\%=((C_{0}-C)/C_{0})\times 100$$

where (C_0_) and (C) represent the initial and time-dependent RB5 concentrations (mg L^−1^), respectively [48].

### 2.5. Total Organic Carbon Analysis

Mineralization performance was evaluated using a Shimadzu TOC-L analyzer (Kyoto, Japan). Total organic carbon (TOC) measurements were conducted before and after photocatalytic treatment [49]. The mineralization efficiency was calculated according to Equation (2):

(2) $$TOC\;Yield\;\%=(C_{0}-C)/C_{0}\times 100$$

where (C_0_) and (C) denote the initial and final TOC concentrations, respectively.

All photocatalytic experiments were performed at least in triplicate, and the reported results represent the average values of independent experiments. The maximum experimental deviation among replicate measurements was within ±5%, indicating good reproducibility of the experimental data.

### 2.6. Kinetic and Arrhenius Analyses Methods

The photocatalytic degradation kinetics of RB5 were evaluated using pseudo-first-order and pseudo-second-order kinetic models. The suitability of each model was assessed based on the correlation coefficient (R^2^) obtained from linear regression analyses.

The temperature dependence of the reaction rate was investigated using the Arrhenius equation:

(3) $$k=A\cdot e^{\frac{-Ea}{RT}}$$

(4) $$\ln k=\ln A-\frac{Ea}{R\cdot T}$$

where (k) is the apparent reaction rate constant (min^−1^), (A) is the pre-exponential factor, (E_a_) is the apparent activation energy (kJ mol^−1^), (R) is the universal gas constant (8.314 J mol^−1^ K^−1^), and (T) is the absolute temperature (K). The activation energy was calculated from the slope of the Arrhenius plot of (ln k) versus (1/T) [39,40,50].

## 3. Results and Discussion

### 3.1. Physicochemical Properties of Chitosan Raw Materials

The physicochemical properties of the chitosan samples used for composite bead preparation are presented in Table 1. Moisture content, ash content, bulk density, and solution viscosity were found to be influenced by both molecular weight and degree of deacetylation (DD).

The moisture content of the chitosan samples varied between 0.098 and 0.593%, while ash content ranged from 0.462 to 1.684%. The lowest ash content was observed for high molecular weight chitosan with 85% DD, indicating a relatively higher purity compared with the commercial chitosan sample. Bulk density values ranged from 0.472 to 1.487 g cm^−3^, with the highest value recorded for the high molecular weight chitosan. The increased density can be attributed to the tighter packing of longer polymer chains, resulting in a more compact structure [33,45,51]. The viscosity of the chitosan solutions varied between 308 and 371 mPa·s (Table 1).

For the samples with 85% DD, viscosity increased with increasing molecular weight, which can be explained by enhanced polymer chain entanglement and stronger intermolecular interactions. Similar relationships between molecular weight and rheological behavior of chitosan solutions have been widely reported in the [34,52,53,54,55,56]. From a bead production perspective, solution viscosity plays a critical role in droplet formation and particle morphology. Solutions with excessively low viscosity may produce irregularly shaped beads, whereas highly viscous solutions can hinder nanoparticle dispersion and mass transfer during gelation. The viscosity range obtained in this study was found to be suitable for producing structurally stable and spherical chitosan/TiO_2_ composite beads.

### 3.2. FTIR Characterization of Chitosan Raw Materials

**Figure 3:**
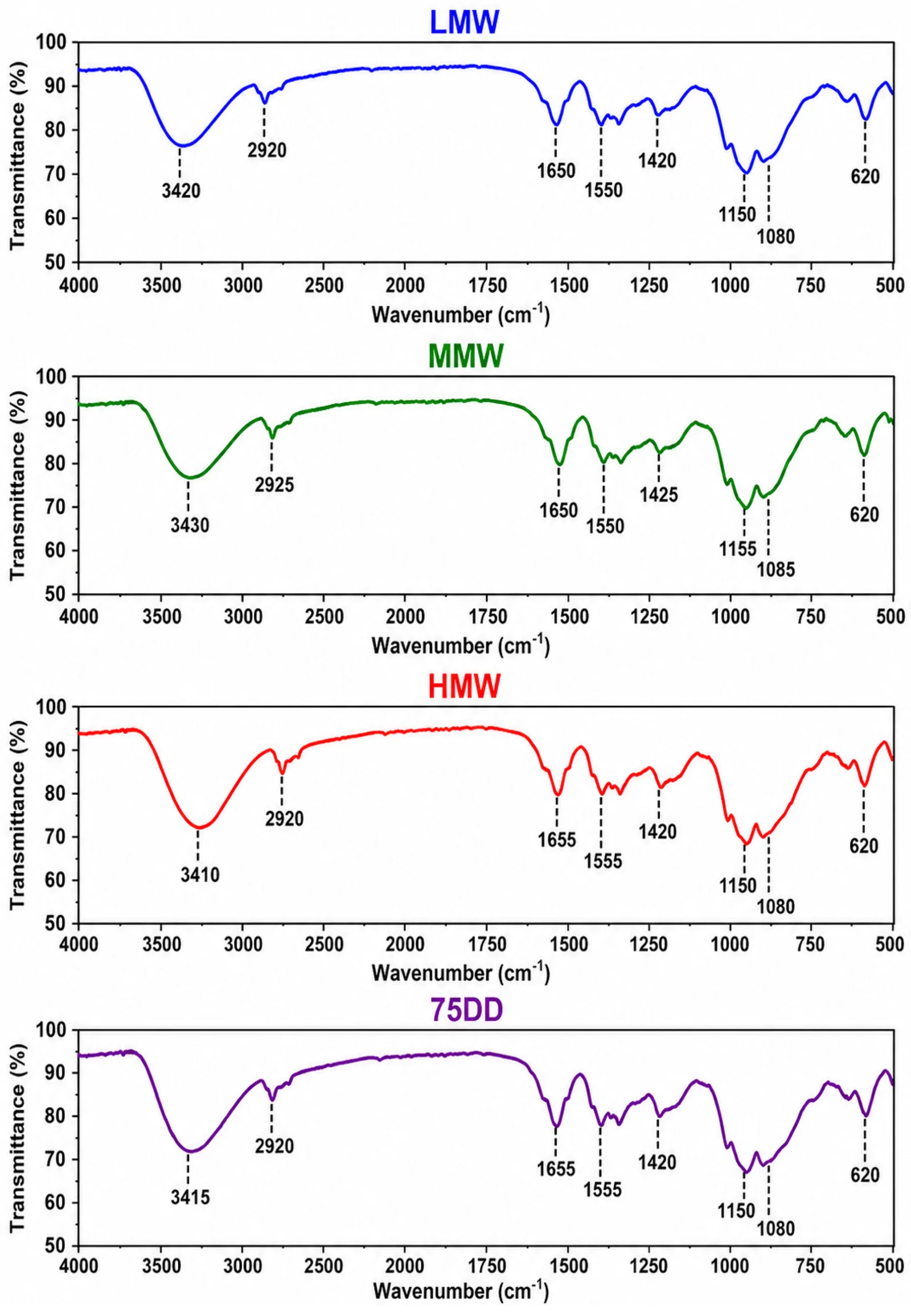
FTIR spectra of chitosan samples with different molecular weights and degrees of deacetylation.

The FTIR spectra of the chitosan samples are presented in Figure 3. All samples exhibited the typical spectral features of chitosan, confirming that the fundamental polysaccharide structure was preserved regardless of molecular weight and degree of deacetylation. A broad absorption band observed within the 3200–3500 cm^−1^ region was assigned to the overlapping stretching vibrations of hydroxyl (O–H) and amino (N–H) groups involved in intermolecular hydrogen bonding. The absorption band around 2920 cm^−1^ corresponded to aliphatic C–H stretching vibrations. Characteristic amide bands were observed near 1650–1655 cm^−1^ (amide I, C=O stretching) and 1550–1590 cm^−1^ (amide II, N–H bending), reflecting the presence of residual N-acetyl groups within the chitosan structure. The bands detected at approximately 1150 and 1080 cm^−1^ were attributed to asymmetric C–O–C glycosidic stretching and C–O stretching vibrations, respectively, confirming the integrity of the polysaccharide backbone. Although the overall spectral profiles were similar, slight variations in peak intensity were observed among the samples. These differences may be associated with variations in molecular weight, degree of deacetylation, and intermolecular hydrogen-bonding interactions. In particular, the relative intensity of the amide bands provides indirect evidence of differences in deacetylation degree, while variations in the broad O–H/N–H absorption region suggest changes in hydrogen-bonding density among the chitosan samples. The observed absorption bands were in good agreement with previously reported FTIR spectra of chitosan materials [37,57,58,59,60] confirming the suitability of the selected raw materials for the preparation of chitosan/TiO_2_ composite photocatalysts.

### 3.3. Physical Characteristics of Chitosan/TiO_2_ Composite Beads

The physical properties of the synthesized chitosan/TiO_2_ composite beads are presented in Table 2. The average particle weight, diameter, volume, and density were significantly influenced by the chitosan characteristics, TiO_2_ loading ratio, and crosslinking treatment.

The particle diameters ranged from 2.09 to 2.85 mm. The smallest diameter was observed for the crosslinked composite bead, P4 ((0.5/1)-(85DD-C)), whereas the largest diameter was obtained for P7 ((0.25/1)-(75DD)). The reduction in particle size after crosslinking can be attributed to the formation of a three-dimensional polymer network, which restricts swelling and promotes a more compact structure [61]. The average particle weight varied between 0.024 and 0.036 g. Although larger particles generally exhibited higher weights, no direct correlation between particle diameter and particle mass was observed, indicating that internal porosity and TiO_2_ distribution also contributed to the overall particle structure.

Density values ranged from 2.975 to 6.067 g cm^−3^. The highest density was obtained for the crosslinked P4 sample, while the lowest density was observed for P7. The substantial increase in density following crosslinking suggests the formation of a more compact polymer matrix with reduced internal void volume. Such structural differences are expected to influence mass transfer properties and photocatalytic performance [61]. Among all synthesized composites, P4 exhibited the smallest diameter and the highest density, indicating that crosslinking promoted the formation of a structurally compact and stable bead architecture. These physical characteristics were later reflected in the photocatalytic performance results, where P4 indicated the highest mineralization efficiency.

### 3.4. Point of Zero Charge (pH_pzc_) Analysis

**Figure 4:**
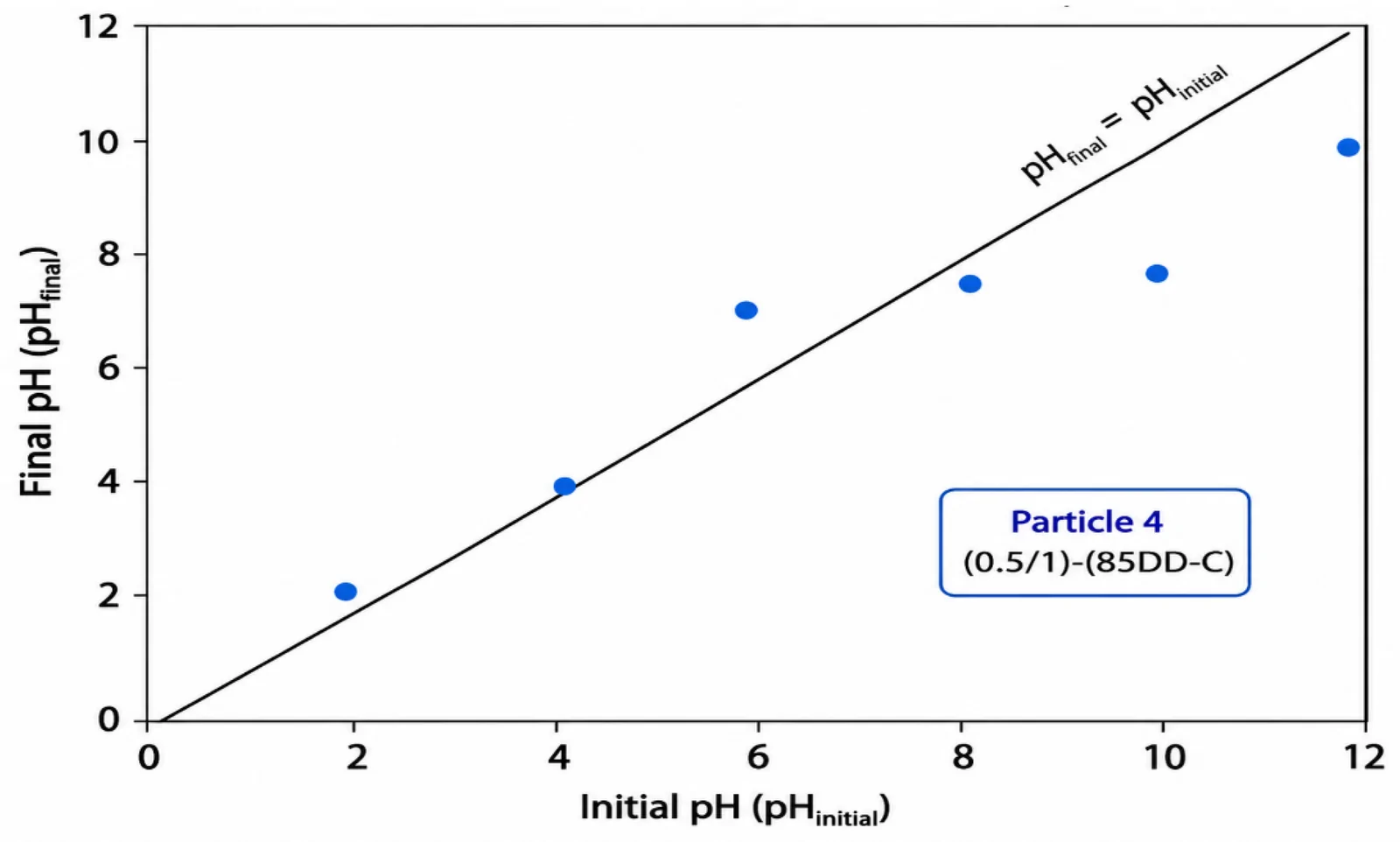
Point of zero charge (pH_pzc_) determination of the selected chitosan/TiO_2_ composite bead (P4).

The pH_pzc_ value of the best-performing composite bead (P4) was determined using the pH drift method [46], and the results are shown in Figure 4. The intersection point between the initial and final pH values was observed at approximately pH 4.8, indicating that the surface of P4 is positively charged below this value and negatively charged above it. The pH_pzc_ value is an important parameter for understanding surface–pollutant interactions during adsorption and photocatalytic degradation processes. At pH values lower than 4.2, protonation of amino groups within the chitosan structure results in a positively charged surface. Since Reactive Black 5 is an anionic dye containing sulfonate groups, strong electrostatic attraction between the dye molecules and the composite surface is expected under acidic conditions. This interaction promotes dye adsorption and facilitates subsequent photocatalytic degradation.

At pH values above the pH_pzc_, deprotonation of surface functional groups leads to the development of a negatively charged surface. Under these conditions, electrostatic repulsion between the composite surface and RB5 molecules may occur, reducing adsorption affinity and consequently decreasing overall removal efficiency [62]. The obtained pH_pzc_ value is consistent with the observed pH-dependent photocatalytic performance, where the highest RB5 removal efficiencies were achieved under acidic conditions. These results suggest that surface charge characteristics play a crucial role in the synergistic adsorption–photocatalytic degradation mechanism of the developed chitosan/TiO_2_ composite beads.

### 3.5. Effect of Composite Composition on Photocatalytic Performance

**Figure 5:**
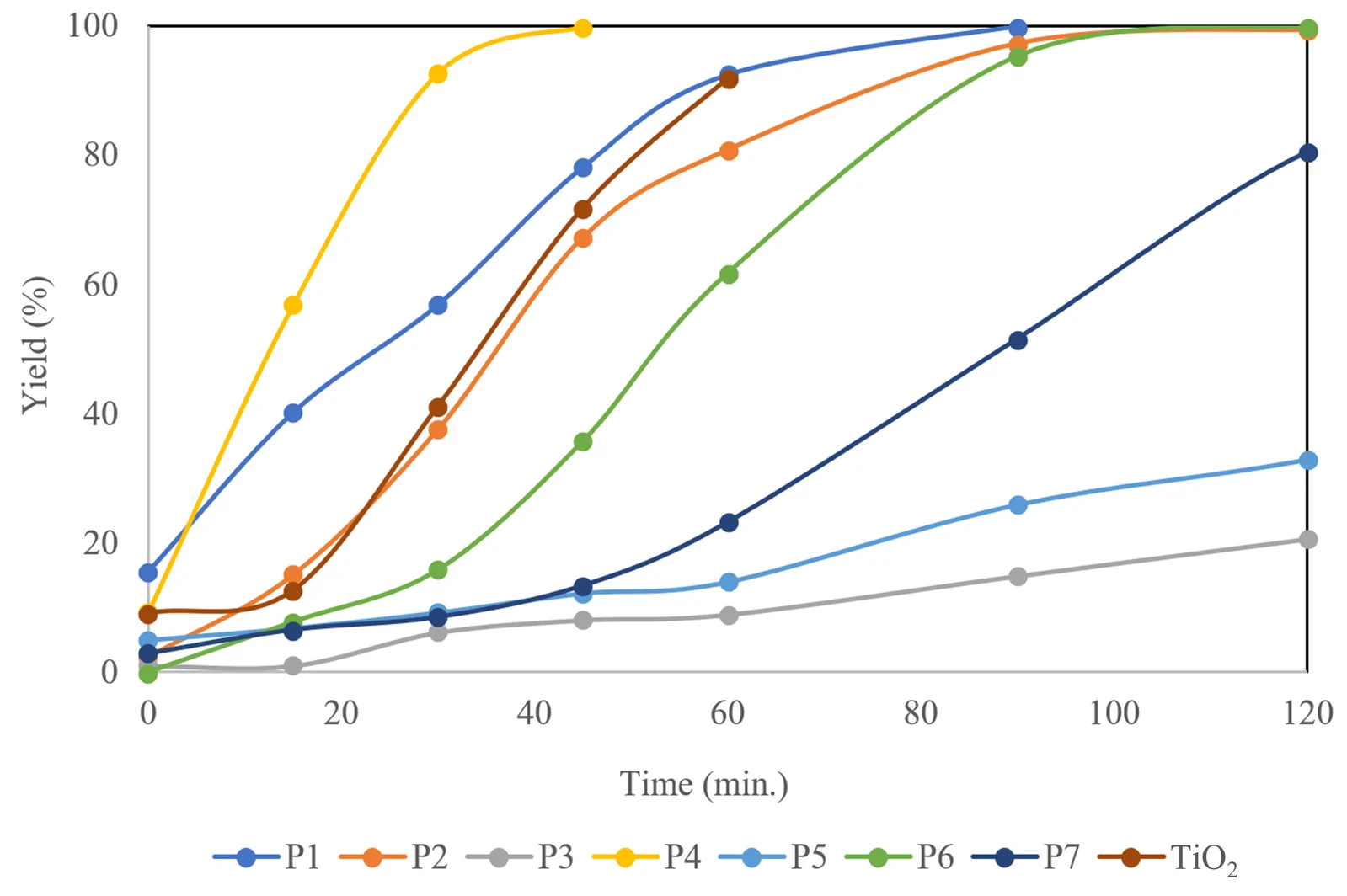
Photocatalytic removal efficiencies of RB5 using chitosan/TiO_2_ composite beads with different compositions.

The photocatalytic performances of the synthesized chitosan/TiO_2_ composite beads were evaluated through the degradation of Reactive Black 5 (RB5) under UV irradiation. The UV–Vis absorption profiles obtained during the photocatalytic experiments are presented in Figure 5.

All composite formulations exhibited measurable photocatalytic activity; however, significant differences in removal performance were observed depending on bead composition. The decrease in absorbance during the reaction indicated the degradation of the chromophoric structure of RB5 and the progressive discoloration of the solution. In general, composite beads prepared using chitosan with a higher degree of deacetylation (85% DD) exhibited superior removal performance compared with those prepared from commercial chitosan with 75% DD [63]. The enhanced performance can be attributed to the greater abundance of amino groups, which promote stronger interactions with both RB5 molecules and TiO_2_ nanoparticles [64].

In the composite structure, TiO_2_ nanoparticles act as photocatalytically active centers, generating electron–hole pairs under UV irradiation and subsequently producing reactive oxygen species such as hydroxyl radicals (•OH) and superoxide radicals (O_2_•^−^). Meanwhile, the chitosan matrix provides adsorption sites that facilitate the accumulation of dye molecules near the photocatalytic surface. The combined UV–Vis, TOC, and FTIR results suggest that adsorption and photocatalytic oxidation jointly contribute to RB5 removal. Although the present results indicate a synergistic interaction between these processes, the exact contribution of each pathway could not be directly quantified within the scope of this study. Although UV–Vis measurements provide valuable information regarding discoloration, they do not indicate the extent of mineralization. Therefore, total organic carbon (TOC) analyses were performed to evaluate the actual degradation performance of the composite beads. The TOC removal efficiencies obtained for different formulations are summarized in Table 3.

The TOC removal efficiencies ranged from 21.32% to 76.23%. The highest mineralization efficiency was achieved with the crosslinked composite bead P4 ((0.5/1)-(85DD-C)), whereas the lowest value was observed for P6. Under identical experimental conditions, TiO_2_ exhibited a TOC removal efficiency of 60.97%, while the optimized crosslinked chitosan/TiO_2_ composite bead (P4) reached 76.23%. Since TOC analysis reflects the mineralization of organic carbon rather than simple dye adsorption, the superior TOC removal achieved by P4 suggests that its enhanced performance cannot be attributed solely to the adsorption capability of chitosan. Instead, the results indicate that the chitosan matrix promotes closer contact between RB5 molecules and photocatalytically active TiO_2_ sites, facilitating subsequent photocatalytic oxidation. Therefore, the improved mineralization performance is more reasonably attributed to the complementary roles of adsorption and photocatalytic oxidation, although the individual contribution of each process was not directly quantified within the scope of the present study.

**Figure 6:**
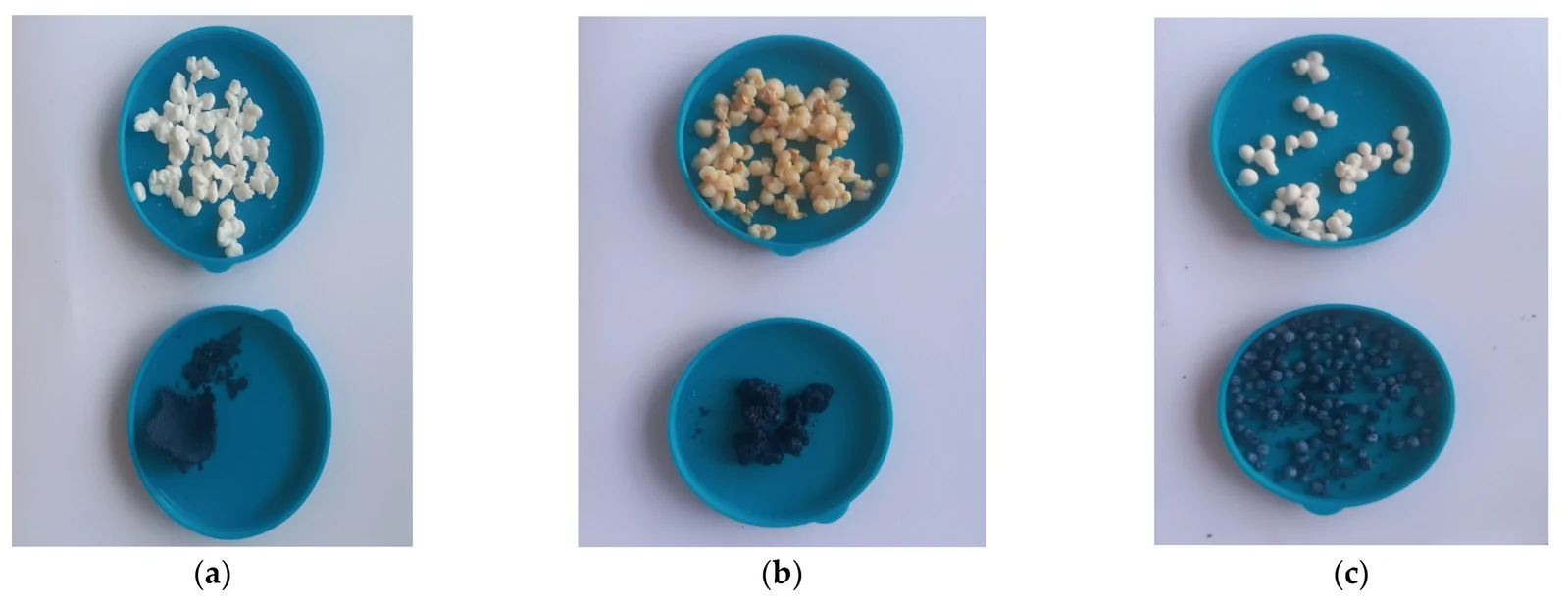
Photographs of chitosan/TiO_2_ composite beads before and after photocatalytic degradation experiments: (**a**) P6, (**b**) P3, and (**c**) P4.

As shown in Figure 6, the superior performance of P4 can be attributed to the optimized combination of crosslinking, chitosan characteristics, and TiO_2_ immobilization. It should be emphasized that the TiO_2_ amount was kept constant in all comparative formulations; therefore, the enhanced performance of P4 cannot be attributed to a higher TiO_2_ loading. Instead, P4 differs primarily from the other formulations by its crosslinked structure. Crosslinking improved the structural compactness and mechanical stability of the polymer matrix while enhancing the retention and dispersion of TiO_2_ particles within the composite beads. In addition, the higher degree of deacetylation provided a greater density of amino groups, increasing the adsorption affinity toward RB5 molecules. However, increasing the chitosan content alone did not result in a proportional improvement in photocatalytic performance, indicating that adsorption was not the sole determining factor. Collectively, these factors enhanced the accessibility of RB5 molecules to photocatalytically active TiO_2_ sites and promoted more efficient photocatalytic oxidation and mineralization.

In contrast, the composites prepared from commercial chitosan with 75% DD exhibited substantially lower mineralization efficiencies, demonstrating the important role of the degree of deacetylation in determining photocatalytic performance. A higher density of amino groups not only enhances dye adsorption but also strengthens the interfacial interactions between TiO_2_ and the polymer matrix, thereby facilitating more efficient photocatalytic oxidation and improving the overall mineralization efficiency.

Compared with suspended TiO_2_, the crosslinked chitosan/TiO_2_ composite beads provide several practical advantages beyond their higher mineralization efficiency. Immobilization of TiO_2_ within the chitosan matrix facilitates catalyst recovery after treatment, minimizes catalyst loss and the potential release of TiO_2_ nanoparticles into the treated water, improves the mechanical stability of the photocatalyst, and provides the potential for regeneration and repeated use. Comprehensive evaluation of long-term recyclability, TiO_2_ leaching, and structural stability after repeated photocatalytic cycles will be the subject of future investigations. These advantages enhance the long-term operational stability, economic feasibility, and environmental sustainability of the photocatalyst for practical wastewater treatment applications. Furthermore, the adsorption capability of chitosan increases the local concentration of RB5 molecules in the vicinity of photocatalytically active TiO_2_ sites, thereby enhancing the probability of subsequent oxidation reactions. Previous studies have also demonstrated that homogeneous TiO_2_ dispersion within polymer matrices improves the accessibility of active sites, suppresses nanoparticle aggregation, promotes charge separation, and consequently enhances photocatalytic efficiency. In addition, photocatalytic degradation proceeds through the generation of electron–hole pairs under UV irradiation, where photogenerated holes directly oxidize adsorbed dye molecules or react with surface hydroxyl groups to generate hydroxyl radicals (•OH), while photogenerated electrons reduce dissolved oxygen to reactive oxygen species such as superoxide radicals (O_2_•^−^), which further contribute to the degradation and mineralization of organic pollutants. The enhanced mineralization observed for P4 is therefore consistent with these generally accepted photocatalytic principles and with the improved interfacial interactions reported for crosslinked chitosan/TiO_2_ composite systems. Although SEM/EDS analyses and direct identification of reactive oxygen species were beyond the scope of the present study, the combined evaluation of FTIR, pHpzc, physical properties, and photocatalytic performance provides indirect evidence supporting the proposed structure–performance relationship and photocatalytic mechanism.

Comparison of the UV–Vis and TOC results further demonstrated that discoloration occurred considerably faster than mineralization, indicating that cleavage of the chromophoric azo groups preceded complete oxidation of the organic structure. Although substantial color removal was achieved for several formulations, TOC removal remained comparatively lower, suggesting the transient formation of intermediate degradation products during photocatalysis. Considering the physical characterization, FTIR spectra, pHpzc analysis, and photocatalytic performance collectively, P4 ((0.5/1)-(85DD-C)) was identified as the optimum composite formulation.

Although direct morphological characterization and reactive species identification were beyond the scope of the present study, the experimental findings provide indirect support for the proposed structure–performance relationship and photocatalytic mechanism. The present findings are consistent with the generally accepted photocatalytic mechanism reported for crosslinked chitosan/TiO_2_ composite systems [65]. Future studies employing SEM/SEM–EDS characterization together with reactive species trapping or advanced spectroscopic techniques would provide further insight into TiO_2_ dispersion, charge separation, and the individual contributions of adsorption and photocatalytic oxidation.

### 3.6. Effect of Initial pH on RB5 Removal

**Figure 7:**
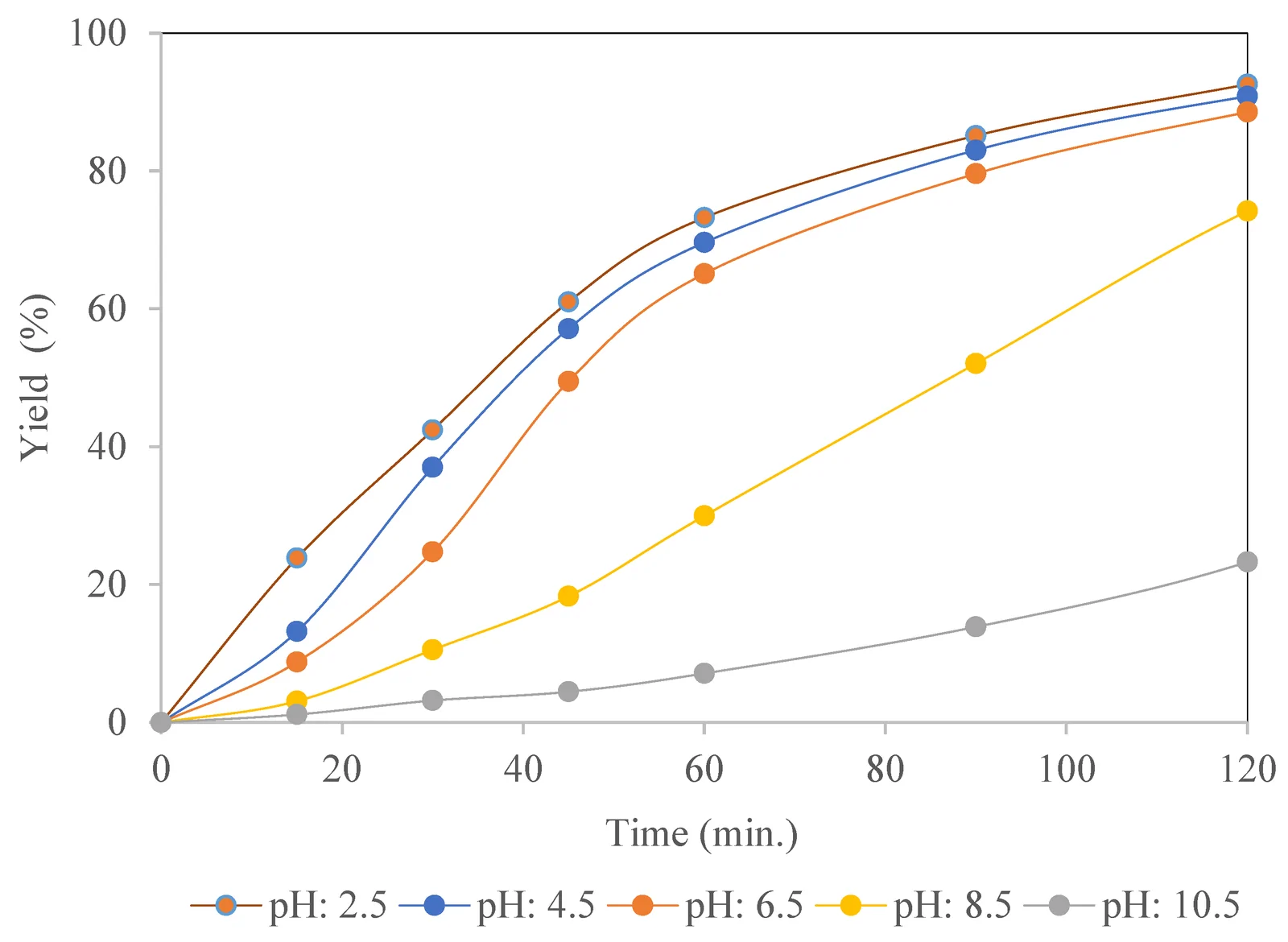
Effect of initial solution pH on RB5 removal efficiency.

The effect of the initial solution pH on the photocatalytic removal of RB5 using the selected chitosan/TiO_2_ composite bead (P4) is presented in Figure 7. The experiments were conducted for 120 min, and the results clearly indicated that pH significantly affected both the degradation rate and the overall removal efficiency. Among the investigated conditions, the highest removal efficiency was achieved at pH 2.5, reaching 92.57% after 120 min of irradiation. At this pH, the removal efficiency increased from 23.85% after 15 min to 60.99% after 45 min, indicating rapid degradation during the initial reaction period. Similarly, high removal efficiencies were obtained at pH 6.5 and 4.5, reaching 90.83% and 88.56%, respectively, after 120 min. In contrast, the removal efficiency decreased to 74.21% at pH 8.5 and further declined to only 23.28% at pH 10.5.

The strong pH dependence of the process can be explained by the surface charge characteristics of the composite beads and the ionic nature of RB5. The pH_pzc_ value of P4 was determined as approximately 4.8. Therefore, at pH values below 4.8, the composite surface becomes positively charged due to protonation of amino groups within the chitosan structure. Since RB5 is an anionic azo dye containing sulfonate groups (–SO_3_^−^), strong electrostatic attraction occurs between the dye molecules and the positively charged composite surface, enhancing adsorption and facilitating subsequent photocatalytic degradation [4,5].

Although electrostatic attraction is expected to decrease at pH values above the pH_pzc_, considerable removal efficiencies were still obtained at pH 4.5 and 6.5. This observation suggests that photocatalytic oxidation played a significant role in the degradation process in addition to adsorption. The presence of TiO_2_ active sites enabled continuous generation of reactive oxygen species, which contributed to RB5 degradation even under conditions where adsorption was less favorable. At alkaline pH values, the removal efficiency decreased markedly. The reduced protonation of amino groups weakened electrostatic interactions between the composite surface and RB5 molecules, while the negatively charged surface generated above the pH_pzc_ promoted electrostatic repulsion. Furthermore, excess hydroxide ions may compete for active sites and influence the formation and utilization of reactive oxygen species, leading to lower photocatalytic activity.

Based on the removal efficiencies obtained after 120 min, the performance followed the order: pH 2.5 > pH 4.5 > pH 6.5 > pH 8.5 > pH 10.5 These findings indicate that both adsorption and photocatalytic oxidation contributed to RB5 removal. The highest efficiency obtained under acidic conditions highlights the importance of electrostatic interactions, whereas the substantial removal observed at near-neutral pH demonstrates the significant contribution of photocatalytic degradation. Therefore, pH 2.5 was selected as the optimum operating condition for subsequent experiments [66,67].

### 3.7. Effect of Composite Bead Dosage on RB5 Removal

**Figure 8:**
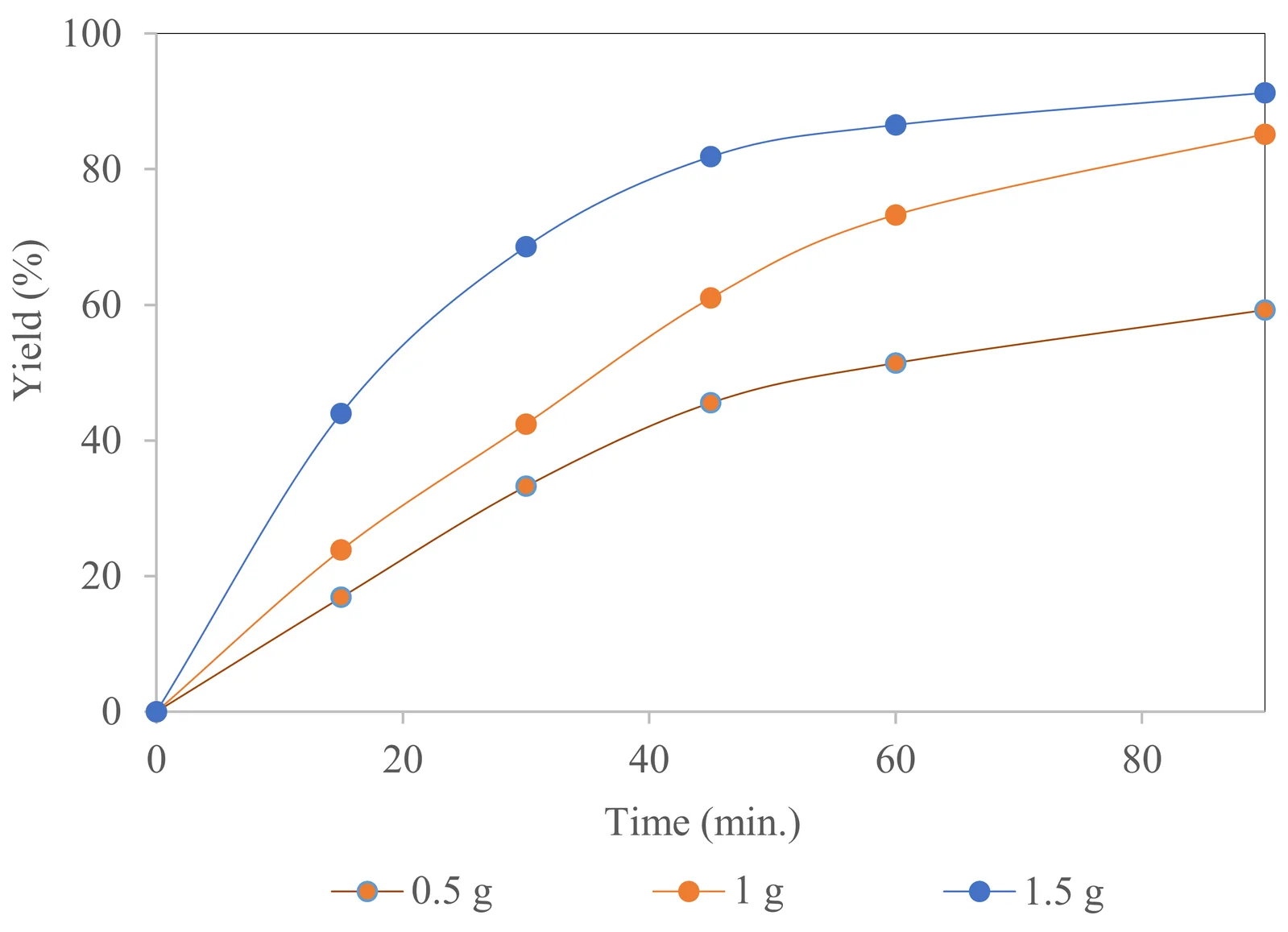
Effect of composite bead dosage on RB5 removal efficiency.

The effect of composite bead dosage on RB5 removal was investigated using 0.5, 1.0, and 1.5 g of the selected chitosan/TiO_2_ composite (P4), and the results are presented in Figure 8. The results clearly indicated that bead dosage significantly influenced the photocatalytic degradation performance.

The lowest removal efficiency was obtained at a dosage of 0.5 g. Under these conditions, RB5 removal reached 16.86% after 15 min, 33.24% after 30 min, and 59.24% after 90 min. The relatively poor performance can be attributed to the limited number of adsorption sites and photocatalytically active TiO_2_ centers available for pollutant degradation.

Increasing the bead dosage to 1.0 g substantially improved the removal efficiency. The degradation efficiency increased to 23.85%, 42.42%, 73.25%, and 85.14% after 15, 30, 60, and 90 min, respectively. The enhanced performance can be attributed to the greater availability of active adsorption sites and photocatalytic centers. Furthermore, the increased amount of catalyst provided a larger illuminated surface area, promoting the generation of hydroxyl radicals (•OH) and other reactive oxygen species responsible for RB5 degradation [3].

The highest removal efficiency was achieved at a dosage of 1.5 g, reaching 44.01% after 15 min and 91.30% after 90 min. The improved performance indicates that the higher catalyst loading increased the number of available active sites for both adsorption and photocatalytic oxidation. Similar trends have been widely reported for heterogeneous photocatalytic systems, where increasing catalyst dosage enhances pollutant degradation up to a certain level.

However, visual observations during the experiments revealed partial fragmentation of the composite beads at the 1.5 g dosage. In addition, a noticeable increase in solution viscosity was observed. These effects may negatively influence long-term operational stability by increasing mass transfer resistance and reducing mixing efficiency. Moreover, fragmented particles may increase turbidity within the suspension, limiting light penetration and potentially reducing the effective utilization of photocatalytically active sites.

Based solely on removal efficiency, the performance followed the order: 1.5 g > 1.0 g > 0.5 g. Nevertheless, considering both degradation performance and operational stability, 1.0 g was selected as the optimum dosage for subsequent experiments. Although the 1.5 g dosage provided slightly higher RB5 removal, the observed bead fragmentation and viscosity increase indicated reduced process robustness. Therefore, 1.0 g represented the most suitable compromise between photocatalytic efficiency and structural stability of the composite beads.

### 3.8. Effect of Initial RB5 Concentration on Photocatalytic Removal

**Figure 9:**
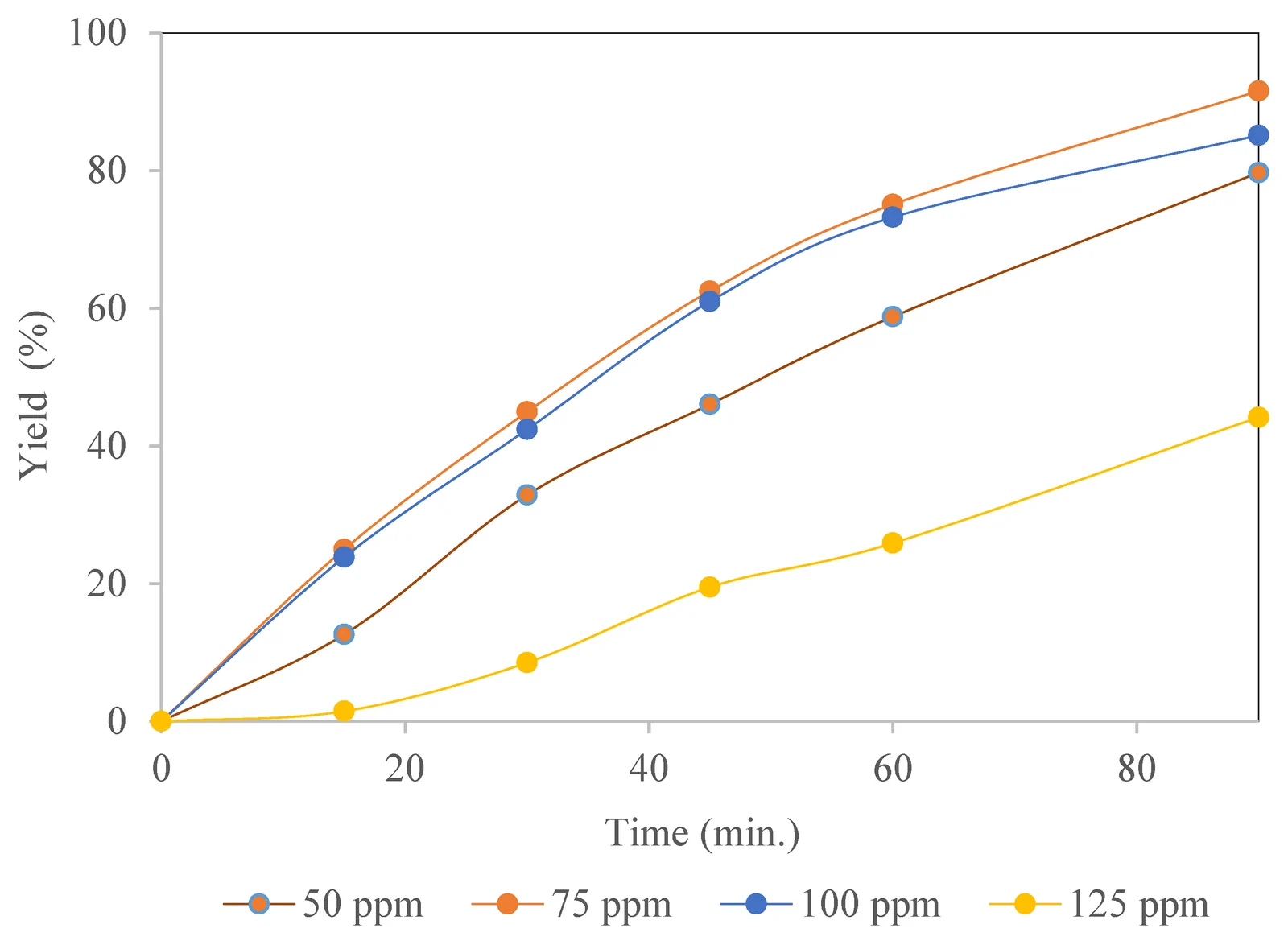
Effect of initial RB5 concentration on photocatalytic removal efficiency.

The effect of the initial RB5 concentration on photocatalytic degradation was investigated using solutions containing 50, 75, 100, and 125 mg L^−1^ RB5, and the results are presented in Figure 9. The obtained results indicated that the initial dye concentration significantly affected both the degradation rate and the final removal efficiency.

Among the investigated concentrations, the highest removal efficiency was achieved at an initial RB5 concentration of 75 mg L^−1^. Under these conditions, the removal efficiency reached 24.99%, 44.96%, 75.11%, and 91.59% after 15, 30, 60, and 90 min, respectively. The superior performance observed at 75 mg L^−1^ suggests that an appropriate balance existed between the number of RB5 molecules present in solution and the available adsorption sites and photocatalytic active centers on the composite beads. As a result, both adsorption and photocatalytic oxidation processes proceeded efficiently.

A similarly high removal efficiency was obtained at an initial concentration of 100 mg L^−1^, reaching 85.14% after 90 min. Although slightly lower than that observed at 75 mg L^−1^, the results indicate that the developed composite beads maintained high photocatalytic activity even under increased pollutant loading. The moderate decrease in removal efficiency can be attributed to increased competition among dye molecules for available active sites.

At the lower concentration of 50 mg L^−1^, the removal efficiency reached 79.73% after 90 min. The lower degradation efficiency compared with the 75 mg L^−1^ system may be associated with the reduced number of dye molecules available for interaction with the adsorption and photocatalytic sites, resulting in less efficient utilization of the active surface.

The lowest performance was observed at an initial concentration of 125 mg L^−1^, where the removal efficiency reached only 44.16% after 90 min. At higher dye concentrations, the number of RB5 molecules in solution increases substantially, leading to competition for adsorption sites and photocatalytic active centers. In addition, the increased color intensity of the solution reduces UV light penetration, thereby decreasing the number of photons reaching the TiO_2_ surface. Consequently, the generation of electron–hole pairs and reactive oxygen species becomes less effective, resulting in lower degradation efficiencies. Furthermore, at elevated pollutant concentrations, adsorption sites become saturated more rapidly, while the amount of hydroxyl radicals (•OH) and other reactive oxygen species generated remains relatively constant. As a result, the oxidative capacity of the system becomes insufficient to completely degrade the increased pollutant load. Similar observations have been reported in previous photocatalytic studies, where increasing dye concentration negatively affected degradation performance due to active-site saturation and reduced light transmission through the reaction medium [6,7,12,68].

Based on the removal efficiencies obtained after 90 min, the performance followed the order: 75 mg L^−1^ > 100 mg L^−1^ > 50 mg L^−1^ > 125 mg L^−1^. Although the highest percentage removal was achieved at 75 mg L^−1^, an initial concentration of 100 mg L^−1^ was selected for subsequent experiments. This selection was made because the system still exhibited high degradation efficiency at a greater pollutant loading, providing a more rigorous assessment of photocatalytic performance and offering conditions that better represent practical wastewater treatment applications.

### 3.9. Effect of Temperature on RB5 Removal

**Figure 10:**
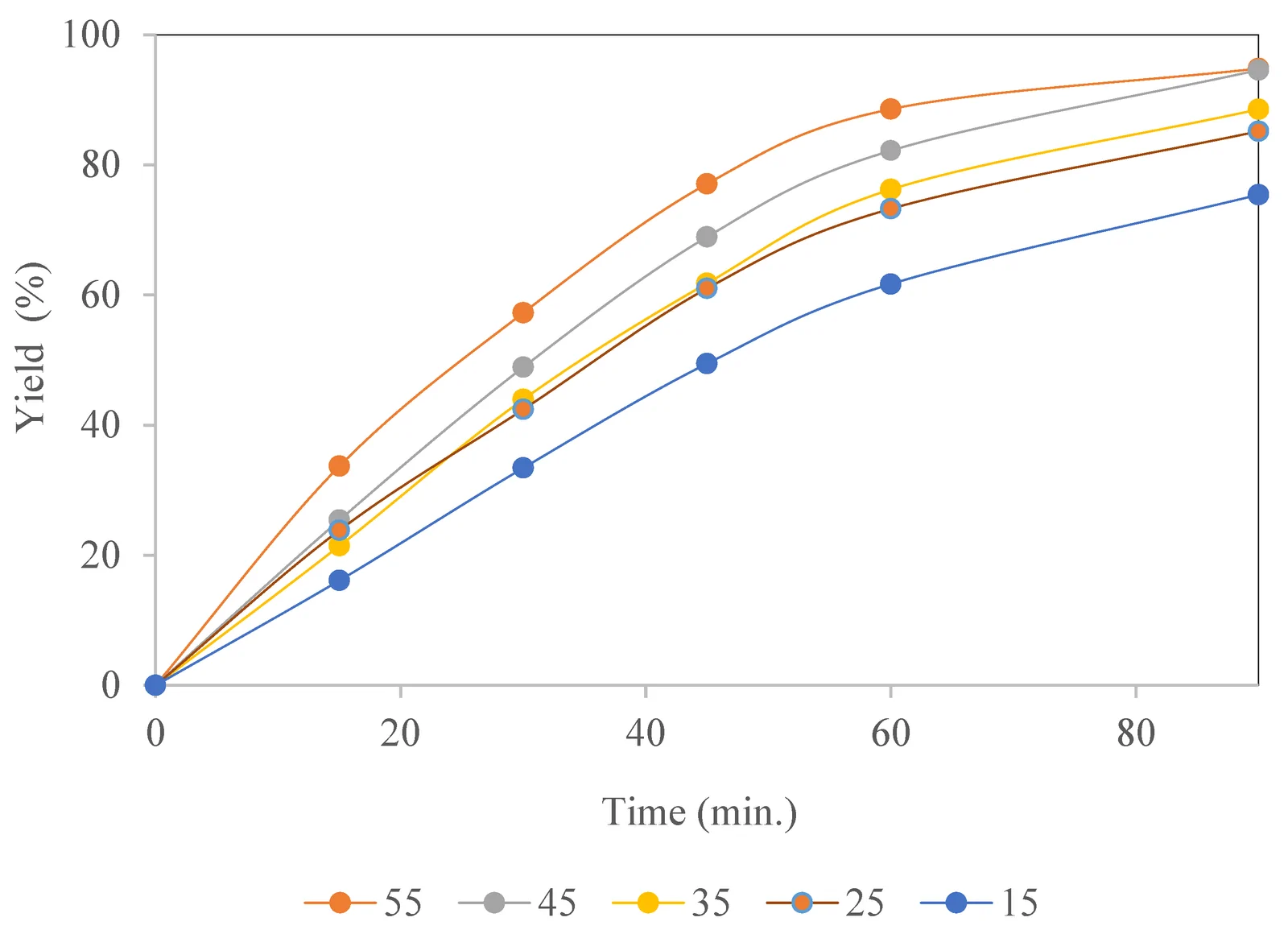
Effect of reaction temperature on RB5 removal efficiency.

The effect of reaction temperature on the photocatalytic degradation of RB5 was investigated at 15, 25, 35, 45, and 55 °C, and the results are presented in Figure 10. The results indicated that temperature significantly influenced both the degradation rate and the final removal efficiency. For all investigated temperatures, the removal efficiency increased with increasing reaction time. However, higher temperatures generally resulted in faster degradation rates and improved final removal efficiencies. The highest RB5 removal efficiency was obtained at 55 °C, reaching 33.69%, 57.27%, 77.08%, and 94.79% after 15, 30, 45, and 90 min, respectively. A very similar performance was observed at 45 °C, where the removal efficiency reached 94.58% after 90 min.

At intermediate temperatures, the removal efficiencies after 90 min were 88.54% and 85.14% for 35 and 25 °C, respectively. Although substantial degradation was achieved under these conditions, the reaction proceeded more slowly than at 45 and 55 °C. The lowest removal efficiency was obtained at 15 °C, reaching only 75.39% after 90 min. The reduced performance at low temperatures can be attributed to slower molecular diffusion, reduced mass transfer rates, and lower reaction rates at the catalyst surface.

The enhancement of photocatalytic performance with increasing temperature may be associated with improved diffusion of RB5 molecules toward active sites and accelerated surface reaction kinetics. Higher temperatures can facilitate interactions between pollutant molecules and photocatalytically active centers, thereby promoting degradation reactions. Similar temperature-dependent behavior has been reported for various photocatalytic systems, where elevated temperatures improve reaction kinetics within a moderate temperature range [69].

The observed trend was also consistent with the kinetic analysis, where the pseudo-first-order rate constants increased progressively with increasing temperature. This behavior confirms that temperature exerts a positive influence on the degradation process and contributes to faster RB5 removal. Based on the removal efficiencies obtained after 90 min, the performance followed the order: 55 °C ≈ 45 °C > 35 °C > 25 °C > 15 °C. Although the highest removal efficiency was obtained at 55 °C, the difference between 45 and 55 °C was relatively small. This observation suggests that the beneficial effect of temperature becomes less pronounced at higher temperatures and that the system approaches a performance plateau. Nevertheless, within the investigated temperature range, 55 °C provided the highest RB5 removal efficiency and was therefore selected as the operating temperature for subsequent kinetic evaluations.

### 3.10. Effect of Operating Parameters on Mineralization Performance

Although UV–Vis analyses provide information regarding dye discoloration, they do not necessarily reflect the complete degradation of organic pollutants. Therefore, total organic carbon (TOC) analyses were performed to evaluate the mineralization efficiency of the photocatalytic process. The TOC removal results obtained under different operating conditions are summarized in Table 4.

The initial solution pH significantly influenced mineralization performance. The highest TOC removal efficiency (76.23%) was achieved at pH 2.5, followed by pH 4.5 (63.07%). As the solution pH increased, TOC removal decreased progressively, reaching 40.24%, 31.71%, and 13.97% at pH 6.5, 8.5, and 10.5, respectively. The enhanced mineralization under acidic conditions can be attributed to the protonation of amino groups within the chitosan structure and the favorable electrostatic attraction between the positively charged composite surface and the anionic RB5 molecules. These interactions facilitate pollutant adsorption and subsequent photocatalytic oxidation, leading to improved mineralization efficiency.

The influence of temperature on TOC removal differed from that observed for color removal. The highest mineralization efficiency was obtained at 25 °C (76.23%), whereas TOC removal decreased to 40.05%, 48.41%, and 59.67% at 35, 45, and 55 °C, respectively. Although higher temperatures accelerated dye discoloration, complete mineralization did not increase proportionally. This finding suggests that elevated temperatures promoted chromophore destruction but may also have favored the accumulation of intermediate oxidation products that were not fully converted into CO_2_ and H_2_O within the experimental period. Initial RB5 concentration also affected mineralization performance. The highest TOC removal was achieved at 100 mg L^−1^ (76.23%), followed by 75 mg L^−1^ (65.02%) and 50 mg L^−1^ (56.03%). At the highest concentration investigated (125 mg L^−1^), TOC removal decreased to 38.57%. The superior performance at 100 mg L^−1^ indicates that this concentration provided a favorable balance between pollutant availability and active-site utilization. At higher concentrations, excessive dye molecules likely occupied adsorption and photocatalytic sites, while increased solution opacity reduced UV light penetration and limited photocatalytic activity.

Although higher temperatures accelerated dye discoloration, complete mineralization did not increase proportionally. This observation suggests that, besides the formation of intermediate oxidation products, elevated temperatures may also have promoted partial deterioration of the crosslinked chitosan matrix, leading to the release of soluble organic species into the aqueous phase. Consequently, these dissolved organic species may have contributed to the measured TOC, resulting in lower apparent mineralization efficiencies despite the enhanced photocatalytic reaction rate.

Composite bead dosage exhibited a pronounced influence on mineralization efficiency. The highest TOC removal (76.23%) was obtained using 1.0 g of composite beads. Reducing the dosage to 0.5 g decreased TOC removal to 27.17%, indicating insufficient active surface area for effective degradation. Interestingly, increasing the dosage to 1.5 g resulted in a further decrease in mineralization efficiency (23.29%).

Experimental observations revealed partial fragmentation of the beads and increased suspension turbidity at this dosage. These effects likely reduced light penetration and hindered mass transfer, thereby limiting photocatalytic activity despite the increased catalyst loading.

A comparison of UV–Vis and TOC results revealed that high discoloration efficiencies did not always correspond to high mineralization efficiencies. In several cases, substantial color removal was achieved while TOC removal remained considerably lower.

This observation indicates that the chromophoric structures of RB5 were readily degraded, whereas complete mineralization of the resulting intermediate products required more extensive oxidation. Therefore, TOC analysis provided a more realistic assessment of the actual degradation performance of the developed photocatalytic system.

Overall, the optimum operating conditions for mineralization were determined as pH 2.5, 100 mg L^−1^ initial RB5 concentration, and 1.0 g composite bead dosage, under which the highest TOC removal efficiency of 76.23% was achieved.

It should be noted that the proposed adsorption–photocatalysis synergy is inferred from the combined evaluation of discoloration (UV–Vis), mineralization (TOC), FTIR characterization, and the observed influence of pH and composite composition on photocatalytic performance. Although these complementary analyses consistently support the proposed mechanism, direct identification of reactive oxygen species or charge transfer pathways was beyond the scope of the present study. Therefore, the mechanistic interpretation presented herein should be considered as a plausible explanation based on the available experimental evidence and the previous literature rather than as direct mechanistic proof.

### 3.11. Kinetic and Arrhenius Analyses

**Figure 11:**
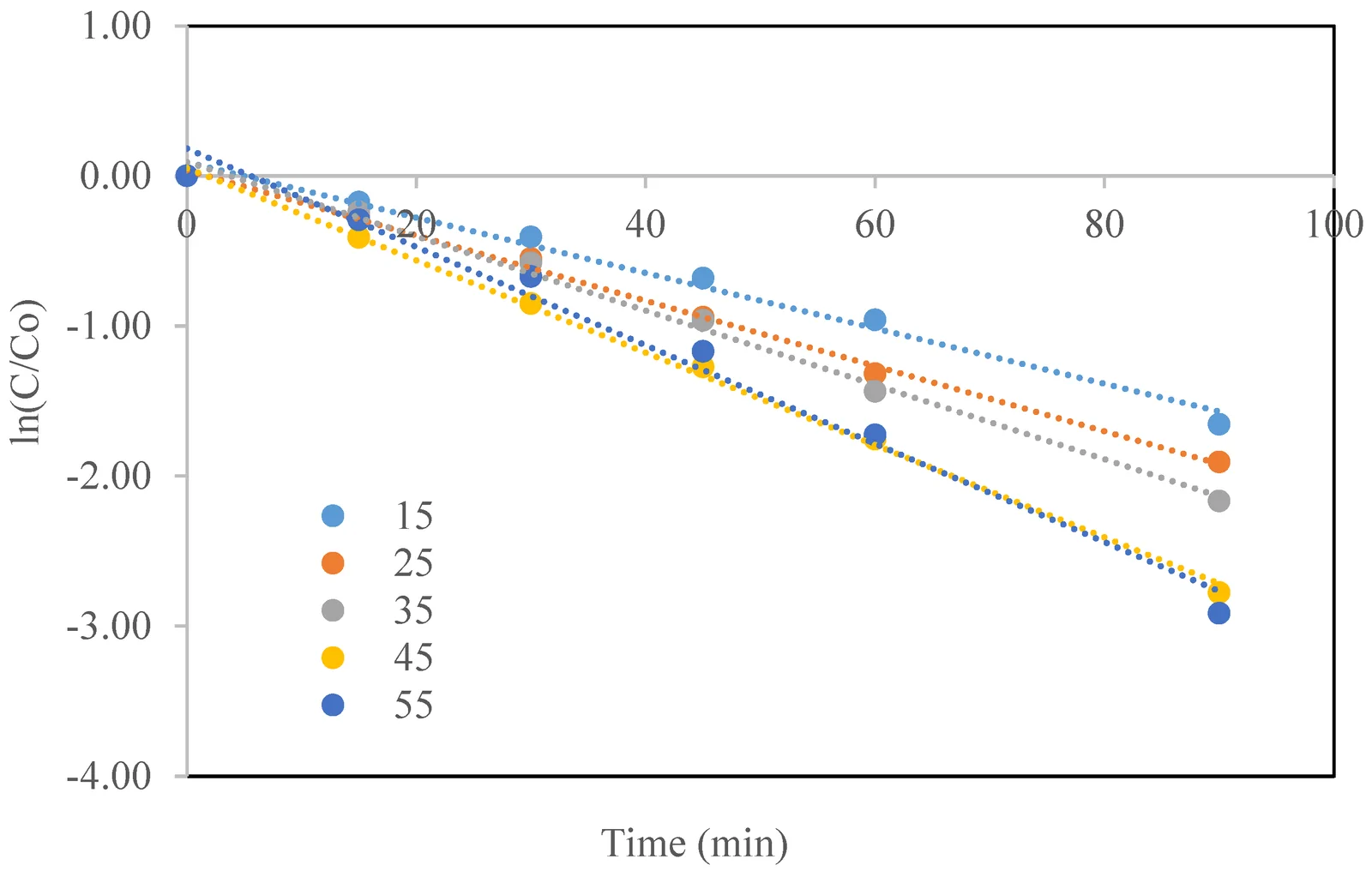
Pseudo-first-order kinetic plots for photocatalytic degradation of RB5 at different temperatures.

**Figure 12:**
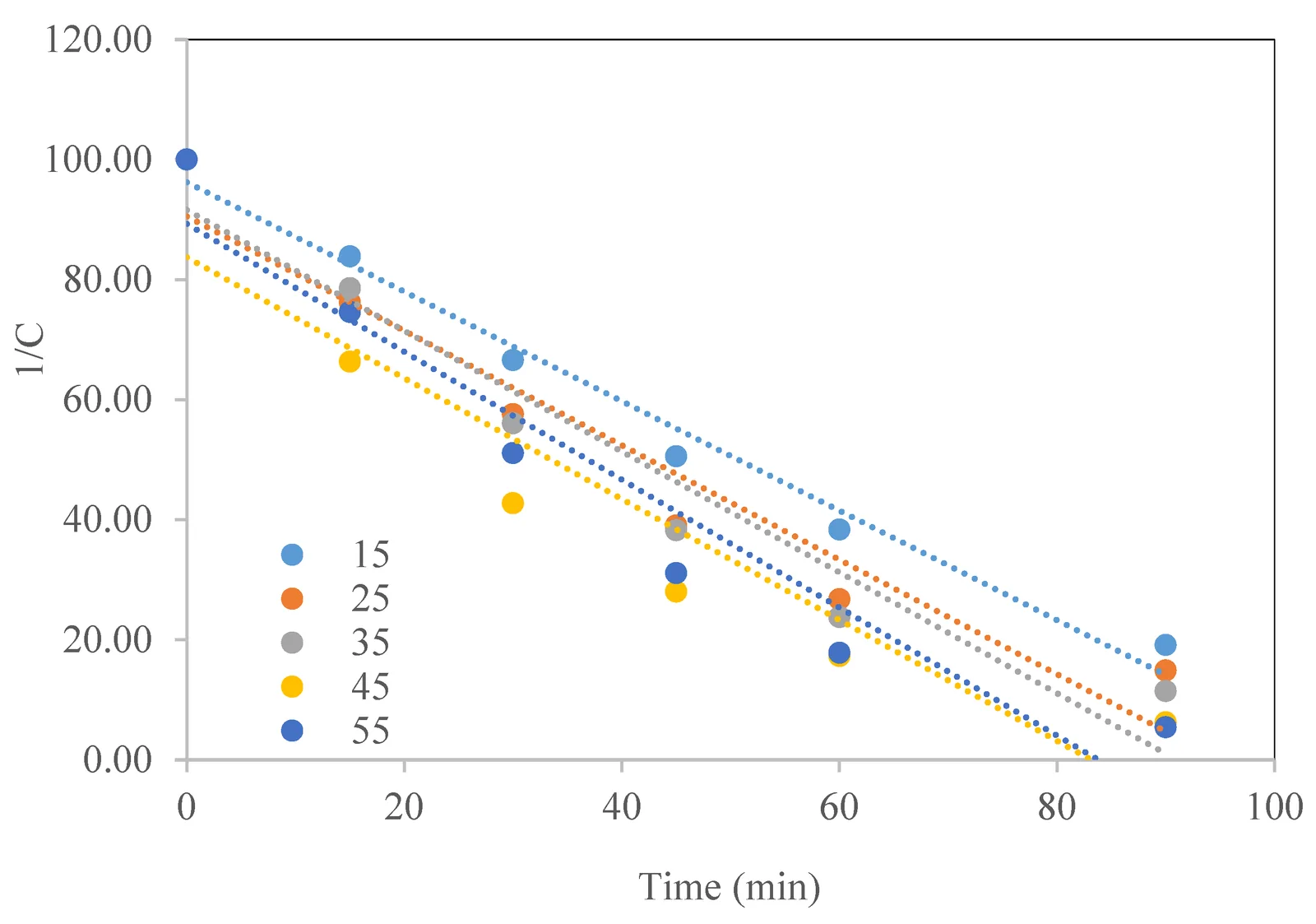
Pseudo-second-order kinetic plots for photocatalytic degradation of RB5 at different temperatures.

To investigate the degradation mechanism of RB5, the experimental data obtained at different temperatures were analyzed using pseudo-first-order (PFO) and pseudo-second-order (PSO) kinetic models. The corresponding linear regression plots are presented in Figure 11 and Figure 12, while the calculated kinetic parameters are summarized in Table 5.

The PFO model exhibited excellent agreement with the experimental data, with correlation coefficients (R^2^) ranging from 0.9841 to 0.9976. The highest correlation coefficient was obtained at 45 °C (R^2^ = 0.9976), whereas the lowest value was observed at 55 °C (R^2^ = 0.9841). Nevertheless, all R^2^ values exceeded 0.98, indicating that the PFO model adequately described the photocatalytic degradation behavior of RB5 throughout the investigated temperature range.

The apparent first-order rate constant (k’) increased progressively with temperature, from 0.0185 min^−1^ at 15 °C to 0.0328 min^−1^ at 55 °C. Intermediate values of 0.0217, 0.0248, and 0.0308 min^−1^ were obtained at 25, 35, and 45 °C, respectively. The increase in reaction rate with temperature suggests enhanced mass transfer, improved diffusion of RB5 molecules toward active sites, and accelerated surface reactions occurring on the photocatalyst surface.

The PSO model also provided reasonable agreement with the experimental data, with R^2^ values ranging from 0.8834 to 0.9826. However, the correlation coefficients obtained for the PSO model were generally lower than those obtained for the PFO model. The highest PSO correlation coefficient was observed at 15 °C (R^2^ = 0.9826), while the lowest value was obtained at 45 °C (R^2^ = 0.8834). These results indicate that the PSO model was less suitable for describing the degradation kinetics of RB5 under the investigated conditions.

A comparison of both kinetic models suggests that the PFO model provides a superior fit to the experimental data. This finding is consistent with the degradation mechanism of heterogeneous photocatalytic systems, where pollutant removal is predominantly governed by the generation of reactive oxygen species and subsequent surface-mediated oxidation reactions. Therefore, the photocatalytic degradation of RB5 by the developed chitosan/TiO_2_ composite beads can be described more accurately using the pseudo-first-order kinetic model.

**Figure 13:**
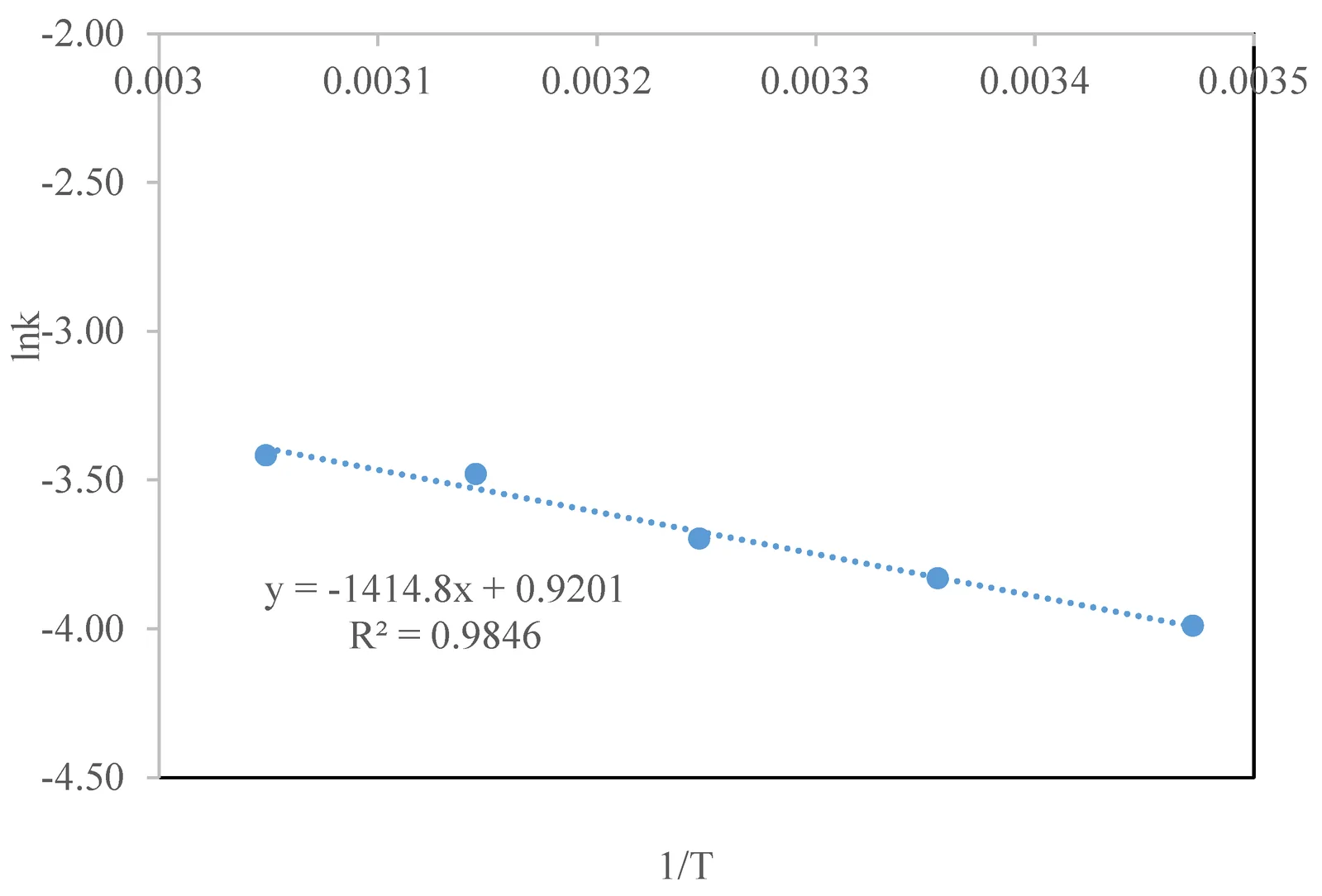
Arrhenius plot obtained from pseudo-first-order rate constants for RB5 photocatalytic degradation.

To evaluate the temperature dependence of the degradation process, the apparent rate constants obtained from the PFO model were further analyzed using the Arrhenius equation. The Arrhenius plot is presented in Figure 13. A strong linear relationship was obtained between ln(k) and 1/T, yielding the equation lnk = −1414.8(1/T) + 0.9201, with a correlation coefficient of R^2^ = 0.9846.

From the slope and intercept of the Arrhenius plot, the apparent activation energy (Ea) and pre-exponential factor (A) were calculated as 11.76 kJ mol^−1^ and 2.51 min^−1^, respectively. The high R^2^ value confirms that the degradation process follows Arrhenius behavior within the investigated temperature range.

The relatively low activation energy indicates that RB5 degradation proceeds with a low energy barrier and can be readily promoted under UV irradiation. Similar activation energy values have been reported for heterogeneous photocatalytic systems, where the reaction is driven not only by thermal activation but also by photoinduced electron–hole generation and subsequent radical-mediated oxidation pathways. Therefore, the obtained Ea value suggests that the degradation process is controlled by a combination of adsorption, charge-transfer reactions, and photocatalytic oxidation rather than by purely thermal effects. Overall, the kinetic and Arrhenius analyses confirm that the developed chitosan/TiO_2_ composite beads exhibit efficient photocatalytic activity toward RB5 degradation and that the reaction rate increases systematically with increasing temperature.

### 3.12. FTIR Analysis of the Composite Beads Before and After Photocatalytic Treatment

**Figure 14:**
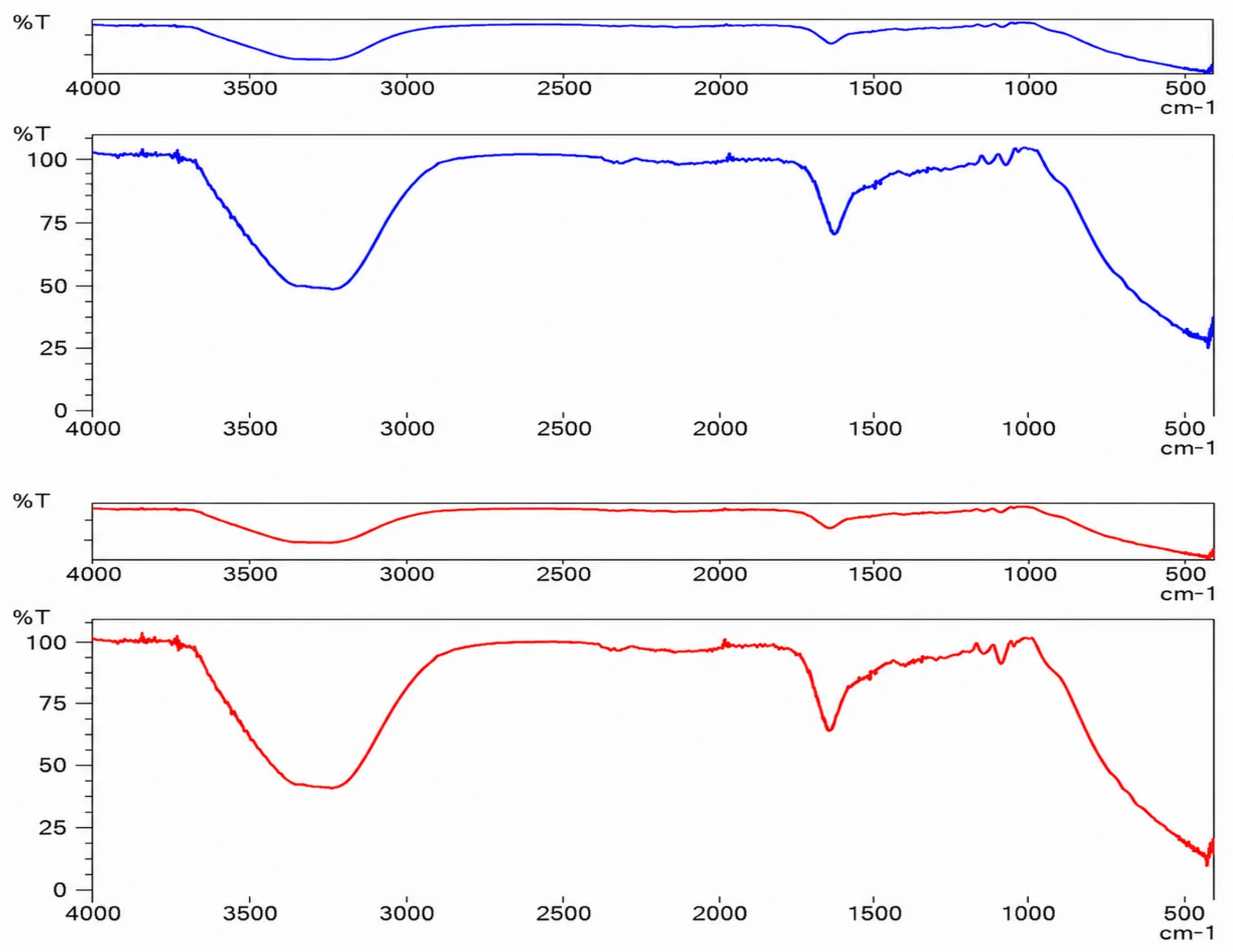
FTIR spectra of the chitosan/TiO_2_ composite bead before and after photocatalytic degradation of RB5.

The FTIR spectra of the chitosan/TiO_2_ composite beads before and after photocatalytic treatment are presented in Figure 14. FTIR analysis was employed to evaluate the structural characteristics of the composite material and to investigate possible interactions between RB5 molecules and the composite surface during the treatment process.

Prior to photocatalytic treatment, the FTIR spectrum exhibited the characteristic absorption bands of both chitosan and TiO_2_. The broad band observed around 3400 cm^−1^ was attributed to the stretching vibrations of hydroxyl (O–H) and amino (N–H) groups present in the chitosan structure. The absorption bands near 2900 cm^−1^ corresponded to aliphatic C–H stretching vibrations. Characteristic amide bands were observed at approximately 1650 cm^−1^ (Amide I, C=O stretching) and 1550 cm^−1^ (Amide II, N–H bending and C–N stretching). In addition, the intense bands located within the 1050–1150 cm^−1^ region were assigned to the glycosidic C–O–C bonds of the chitosan backbone. The bands observed in the low-wavenumber region (450–550 cm^−1^) were attributed to Ti–O–Ti and Ti–O vibrations, confirming the successful incorporation of TiO_2_ within the chitosan matrix. Following photocatalytic treatment, noticeable changes were observed in several regions of the spectrum.

Variations in the intensity of the bands around 1600 cm^−1^ suggest interactions between RB5 molecules and functional groups present on the composite surface. Furthermore, bands appearing or becoming more pronounced in the 1450–1500 cm^−1^ region may be associated with aromatic ring vibrations and residual dye-related structures. Similarly, changes observed in the 1200–1250 cm^−1^ region can be attributed to the presence of sulfonate-containing functional groups originating from RB5 molecules. The broad O–H/N–H band around 3400 cm^−1^ also exhibited slight shifts and intensity variations after treatment.

These changes indicate possible hydrogen-bonding interactions between the amino and hydroxyl groups of chitosan and the functional groups of RB5 molecules. In addition, the broadening observed around 1050 cm^−1^ suggests overlapping contributions from chitosan and dye-derived functional groups adsorbed on the composite surface. The FTIR results indicate that RB5 molecules interacted with the functional groups present on the chitosan/TiO_2_ composite surface during the treatment process. When considered together with the UV–Vis and TOC results, these findings suggest that adsorption played an important role in the initial capture of dye molecules, while photocatalytic oxidation contributed to their subsequent degradation. Therefore, the overall removal mechanism can be attributed to the synergistic contribution of adsorption and photocatalytic processes. Overall, FTIR analysis confirms the preservation of the composite structure after treatment and provides evidence for surface interactions between RB5 molecules and the chitosan/TiO_2_ composite beads, supporting the proposed adsorption-assisted photocatalytic degradation mechanism.

## 4. Conclusions

In this study, chitosan/TiO_2_ composite beads with different compositions were successfully synthesized and evaluated for the photocatalytic removal of Reactive Black 5 (RB5) from aqueous solutions. The results indicated that the physicochemical properties of chitosan, particularly the degree of deacetylation and crosslinking treatment, significantly influenced the structural characteristics and photocatalytic performance of the prepared composites. Among the synthesized materials, the crosslinked composite bead prepared using chitosan with an 85% degree of deacetylation (Particle 4) exhibited the highest mineralization performance, achieving 76.23% TOC removal. The superior performance of this material was attributed to its enhanced structural stability, higher density, and improved retention of TiO_2_ particles within the chitosan matrix.

Operational parameter studies revealed that pH, catalyst dosage, initial dye concentration, and temperature played critical roles in the photocatalytic degradation process. The highest RB5 removal efficiency (approximately 95%) was obtained under acidic conditions (pH 2.5), using 1.5 g of composite beads, an initial RB5 concentration of 75 mg L^−1^, and a reaction temperature of 55 °C. However, due to bead fragmentation and viscosity-related operational limitations observed at higher catalyst dosages, 1.0 g was selected as the optimum dosage for subsequent experiments. FTIR analyses suggested the interaction between RB5 molecules and the composite surface, indicating that adsorption and photocatalytic oxidation jointly contributed to the overall removal mechanism.

Kinetic evaluations showed that the degradation process was best described by the pseudo-first-order kinetic model, with correlation coefficients ranging from 0.9841 to 0.9976. Furthermore, Arrhenius analysis yielded an apparent activation energy of 11.76 kJ mol^−1^, indicating that the degradation process proceeds with a relatively low energy barrier and that the reaction rate increases with increasing temperature.

The main findings of this study can be summarized as follows:

- Crosslinked chitosan/TiO_2_ composite beads exhibited significantly enhanced photocatalytic and mineralization performance compared with non-crosslinked composites.
- The highest TOC removal efficiency (76.23%) was achieved using Particle 4, indicating effective mineralization of RB5.
- RB5 removal was strongly affected by solution pH, catalyst dosage, initial dye concentration, and reaction temperature.
- FTIR analyses supported the synergistic contribution of adsorption and photocatalytic oxidation mechanisms during the degradation process.
- The degradation kinetics followed a pseudo-first-order model more closely than a pseudo-second-order model.
- The low apparent activation energy (11.76 kJ mol^−1^) indicated the favorable kinetic behavior of the developed photocatalytic system.

Overall, the developed chitosan/TiO_2_ composite beads represent a sustainable and environmentally friendly photocatalytic material with considerable potential for the treatment of dye-contaminated wastewater and advanced water purification applications.

Preliminary recycling experiments performed at 25 °C over three consecutive cycles demonstrated that the crosslinked chitosan/TiO_2_ composite beads retained more than 98% of their initial photocatalytic performance, indicating excellent operational stability and further supporting their potential for practical wastewater treatment applications.

Although the present findings support the proposed complementary roles of adsorption and photocatalytic oxidation, further investigations involving long-term cycling performance, TiO_2_ leaching analysis, post-reaction structural characterization, reactive species trapping experiments, and advanced spectroscopic techniques are currently in progress to provide a more comprehensive understanding of the durability, stability, and photocatalytic mechanism of the developed composite system.

## Figures and Tables

**Table 1:** Physicochemical properties of the chitosan samples used for composite bead preparation.

Chitosan Sample	Moisture (%)	Ash Content (%)	Bulk Density (g cm^−3^)	Viscosity (mPa·s)
LMW (85% DD)	0.098 ± 0.003	1.327 ± 0.051	1.421 ± 0.058	308 ± 9
MMW (85% DD)	0.367 ± 0.012	1.114 ± 0.043	0.811 ± 0.029	326 ± 11
HMW (85% DD)	0.542 ± 0.018	0.462 ± 0.014	1.487 ± 0.063	371 ± 13
Commercial chitosan (75% DD)	0.593 ± 0.021	1.684 ± 0.069	0.472 ± 0.017	344 ± 10

**Table 2:** Physical characteristics of synthesized chitosan/TiO_2_ composite beads (particle weight, diameter, volume, and density).

Sample	Weight (g)	Diameter (mm)	Volume (cm^3^)	Density (g cm^−3^)
P1 (1/1)-(85DD)	0.024 ± 0.002	2.29 ± 0.11	0.0063	3.817
P2 (0.5/1)-(85DD)	0.029 ± 0.002	2.31 ± 0.12	0.0065	4.493
P3 (0.25/1)-(85DD)	0.029 ± 0.005	2.36 ± 0.18	0.0069	4.214
P4 (0.5/1)-(85DD-C)	0.029 ± 0.001	2.09 ± 0.18	0.0048	6.067
P5 (1/1)-(75DD)	0.032 ± 0.001	2.32 ± 0.16	0.0065	4.894
P6 (0.5/1)-(75DD)	0.036 ± 0.004	2.52 ± 0.10	0.0084	4.296
P7 (0.25/1)-(75DD)	0.036 ± 0.003	2.85 ± 0.06	0.0121	2.975

**Table 3:** TOC removal efficiencies and mineralization performances of chitosan/TiO_2_ composite beads during RB5 degradation.

Sample	Initial TOC (mg L^−1^)	Final TOC (mg L^−1^)	TOC Removal (%)
P1 (1/1)-(85DD)	2.62	1.36	48.21
P2 (0.5/1)-(85DD)	2.68	1.54	42.57
P3 (0.25/1)-(85DD)	2.66	1.29	51.43
P4 (0.5/1)-(85DD-C)	2.65	0.63	76.23
P5 (1/1)-(75DD)	2.68	1.79	33.28
P6 (0.5/1)-(75DD)	2.72	2.14	21.32
P7 (0.25/1)-(75DD)	2.62	1.88	28.09
TiO_2_	2.69	1.05	60.97

**Table 4:** TOC removal efficiencies obtained under different operating conditions.

Parameter	Condition	TOC Removal (%)
pH	2.5	76.23
	4.5	63.07
	6.5	40.24
	8.5	31.71
	10.5	13.97
Temperature	25 °C	76.23
	35 °C	40.05
	45 °C	48.41
	55 °C	59.67
Initial RB5 concentration	50 mg L^−1^	56.03
	75 mg L^−1^	65.02
	100 mg L^−1^	76.23
	125 mg L^−1^	38.57
Bead dosage	0.5 g	27.17
	1.0 g	76.23
	1.5 g	23.29

**Table 5:** Kinetic parameters obtained from pseudo-first-order and pseudo-second-order models for RB5 photocatalytic degradation.

Model	Temperature (°C)	Kinetic Equation	Rate Constant	R^2^
Pseudo-first-order	15	ln(C/C_0_) = −0.0185t + 0.0916	0.0185	0.9861
	25	ln(C/C_0_) = −0.0217t + 0.0373	0.0217	0.9966
	35	ln(C/C_0_) = −0.0248t + 0.0933	0.0248	0.9934
	45	ln(C/C_0_) = −0.0308t + 0.0530	0.0308	0.9976
	55	ln(C/C_0_) = −0.0328t + 0.1821	0.0328	0.9841
Pseudo-second-order	15	1/C = −0.9119t + 96.215	0.9119	0.9826
	25	1/C = −0.9536t + 90.536	0.9536	0.9356
	35	1/C = −1.0066t + 91.610	1.0066	0.9411
	45	1/C = −1.0071t + 83.717	1.0071	0.8834
	55	1/C = −1.0654t + 89.285	1.0654	0.9280

## Data Availability

The data supporting the findings of this study are available from the corresponding author upon reasonable request.
